# Transposon Defense by Endo-siRNAs, piRNAs and Somatic pilRNAs in *Drosophila*: Contributions of Loqs-PD and R2D2

**DOI:** 10.1371/journal.pone.0084994

**Published:** 2014-01-13

**Authors:** Milijana Mirkovic-Hösle, Klaus Förstemann

**Affiliations:** Gene Center and Department of Biochemistry, Ludwig-Maximilians-Universität, Munich, Germany; The John Curtin School of Medical Research, Australia

## Abstract

Transposable elements are a serious threat for genome integrity and their control via small RNA mediated silencing pathways is an ancient strategy. The fruit fly *Drosophila melanogaster* has two silencing activities that target transposons: endogenous siRNAs (esiRNAs or endo-siRNAs) and Piwi-interacting small RNAs (piRNAs). The biogenesis of endo-siRNAs involves the Dicer-2 co-factors Loqs-PD, which acts predominantly during processing of dsRNA by Dcr-2, and R2D2, which primarily helps to direct siRNAs into the RNA interference effector Ago2. Nonetheless, loss of either protein is not sufficient to produce a phenotype comparable with a *dcr-2* mutation. We provide further deep sequencing evidence supporting the notion that R2D2 and Loqs-PD have partially overlapping function. Certain transposons display a preference for either dsRBD-protein during production or loading; this appeared to correlate neither with overall abundance, classification of the transposon or a specific site of genomic origin. The endo-siRNA biogenesis pathway in germline operates according to the same principles as the existing model for the soma, and its impairment does not significantly affect piRNAs. Expanding the analysis, we confirmed the occurrence of somatic piRNA-like RNAs (pilRNAs) that show a ping-pong signature. We detected expression of the Piwi-family protein mRNAs only barely above background, indicating that the somatic pilRNAs may arise from a small sub-population of somatic cells that express a functional piRNA pathway.

## Introduction

The small RNA silencing system is essential for gene regulation, antiviral defense and the control of transposable elements in the genome [1], [2]. In *Drosophila*, these diverse tasks are distributed among different branches, with miRNAs serving to regulate endogenous gene expression, siRNAs mediating antiviral protection and the mostly germline restricted piRNAs preventing the spread of selfish genetic elements [3]. The repression exerted by miRNAs and antiviral as well as experimental siRNAs occurs at the post-transcriptional level. In contrast, piRNAs mediate both transcriptional and post-transcriptional silencing of their targets. Recent studies indicate that an additional siRNA class with endogenous origin can also repress transposons [4]–[8]. The siRNA and piRNA pathways thus target transposons jointly in the germline, whereas somatic repression is mediated predominantly by the siRNA pathway.

Although the biogenesis of transposon-targeting piRNAs and endo-siRNAs is mechanistically quite distinct, transcripts in sense- and antisense-orientation are required in both cases. This potentially leads to a competition for precursor molecules in the germline, where both pathways are active concomitantly. While it has been previously demonstrated that the piRNA system is unperturbed in *dcr-2* mutant flies [7], it is possible that the endo-siRNA system has a distinct mode of operation in soma vs. germline. During endo-siRNA biogenesis, the RNaseIII enzyme Dicer-2 associates with two distinct dsRNA-binding-domain containing proteins (dsRBPs, Loqs-PD and R2D2) which act predominantly during dsRNA processing and Ago2-loading, respectively [9]–[11]. This biogenesis model has been derived from experiments performed with whole flies [12], exclusively somatic tissues [4] or cultured somatic cells [9], [10], [13]. We examined the contribution of R2D2 and Loqs-PD genetically and compared somatic tissues with ovaries (as a surrogate for germline). We confirm that piRNA biogenesis is unaffected by the presence or absence of either dsRBP protein. Furthermore, we demonstrate that endo-siRNA biogenesis occurs analogously in germline and soma and that there appears to be a certain extent of redundancy between Loqs-PD and R2D2 for Ago2-loading. Finally, we substantiate existing evidence for the occurrence of somatic pilRNAs. They show a ping-pong signature in their sequences and are of rather low abundance, indicating that there may be a small subset of somatic cells that express a complete and active piRNA pathway.

## Results

### Deep sequencing of somatic and germline RNA samples

To further elucidate the contributions of R2D2 and Loqs-PD in transposon defense we took a genetic approach. Since the *loqs* gene generates several isoforms with specialized functions (PA and PB interacting with Dcr-1 during miRNA biogenesis, PD interacting with Dcr-2 in siRNA biogenesis and PC with unknown function) [9], [10], [13], we employed flies with a full deletion of the *loqs* locus that carried a transgene to re-introduce a Loqs-PB cDNA and restore miRNA biogenesis [14]. These flies therefore lack the Loqs isoforms PA, PC and PD. Since Loqs-PB can fully complement the miRNA phenotype and the expression of Loqs-PC is potentially negligible, we will refer to these flies as *loqs-D* mutants in the remainder of this manuscript for simplicity. For *r2d2*, a mutant allele generated by imprecise excision of a P-element was used (*r2d2^1^* [15]). Since our focus was on transposable element regulation and the two mutant fly strains had different genetic backgrounds, we performed one round of back-crossing with *w^1118^* (see materials and methods in File S1 as well as Figure S1 to S4 in File S1 for crossing and validation schemes). We note that a single round of back-crossing reduces but certainly does not abolish differences in transposon content and/or localization between the strains. Given the mobile nature of selfish genetic elements, however, a completely homogeneous background is likely never achievable. The back-crossed *loqs^ko^; P{Loqs-PB}* and *r2d2^1^* mutant fly stocks were maintained as balanced stocks (*CyO*) from which homozygous mutant animals were selected for RNA isolation and deep sequencing. Heterozygous controls were obtained by crossing balanced flies with *w^1118^* and selecting the non-*Cy* offspring thus carrying a mutant and a wild-type, rather than a balancer, chromosome. As a somatic sample, we prepared RNA from the head and thorax portion of female flies while dissected ovaries were used as a predominantly germline derived sample.

To differentiate between Ago2-loaded and other small RNAs, we made use of the fact that the 3’-terminal nucleotide of Piwi-/Aub-/Ago3- as well as Ago2-loaded small RNAs is 2’-*O*-methyl modified [16], [17]. This modification renders the small RNAs resistant to oxidation of vicinal diols with sodium periodate and subsequent β-elimination that will shorten the un-modified RNAs by one nucleotide and prevent them from participating in the 3’-end ligation reaction required for deep sequencing library generation. The technique is highly efficient and specific since the β-elimination resistant small RNAs essentially disappear in libraries prepared from *ago2* null mutant flies [4]. Although oxidation alone would suffice for selective sequencing of Ago2 and Piwi-clade protein loaded small RNAs, we included the β-elimination step to allow for visual control of successful and complete oxidation by electrophoresis and staining of the 30 nt long 2S rRNA (see Figure S5 in File S1 for gel images). Thus, the contribution of R2D2 and Loqs-PD to the processing of a certain small RNA species can be revealed by reduction in both untreated and β-eliminated libraries, while a selective contribution to the loading step is evident by reduction only in the β-eliminated libraries. Untreated and β-eliminated RNA samples were fractionated on acrylamide-urea gels and the ∼17-28 nt long RNAs were excised using the endogenous 2S rRNA as a marker. After adapter ligation and PCR amplification the libraries were sequenced on the Illumina GAIIx platform. Table 1 summarizes the total number of reads, the proportion of reads matching the *Drosophila* genome and the amount of reads that mapped to a collection of transposon sequences (EMBL/FlyBase collection) or miRNAs. No mismatches were allowed during mapping of the reads. A first analysis of all transposon-matching endo-siRNAs and all miRNAs in the somatic RNA samples revealed that as expected in control animals, miRNAs are sensitive and siRNAs resistant to β-elimination. In *r2d2* mutants, the endo-siRNAs have become sensitive to the chemical treatment but are still distinguishable from miRNAs in this respect. In the case of *loqs-D* mutants, transposon-matching endo-siRNAs have also become somewhat sensitive to β-elimination but less than in the case of *r2d2* (Figure S6 in File S1). In all cases, the difference between endo-siRNAs and miRNAs demonstrates that the oxidation reaction has not been limiting. Note that certain *Drosophila* miRNAs are also partially loaded into Ago2, thus explaining the heterogeneous extent of their susceptibility to β-elimination [18], [19].

**Table 1:** Analysis of deep sequencing libraries generated in this study.

SOMA					
Library	β-eliminated	Total no of insert11–28 nt	Insertsmatchingthe genome(% of total)	InsertsmatchingmiRNAs(% of genomematching)	Insertsmatchingtransposons(% of genomematching)
*loqs-D*/+	–	9546079	7082781 **(74.2)**	3421132 **(48.3)**	36408 **(0.5)**
*loqs-D*/+	+	3682719	2253071 **(61.2)**	464462 **(20.6)**	114366 **(5.1)**
*loqs-D*/*loqs-D*	–	22704677	18806689 **(82.8)**	10946243 **(58.2)**	80909 **(0.4)**
*loqs-D*/*loqs-D*	+	3103232	1348791 **(43.5)**	415769 **(30.8)**	73543 **(5.5)**
*r2d2^1^*/+	–	20954822	15961906 **(76.2)**	7213032 **(45.2)**	118789 **(0.7)**
*r2d2^1^*/+	+	4906737	2444080 **(49.8)**	568163 **(23.2)**	438137 **(17.9)**
*r2d2^1^*/ *r2d2^1^*	–	4333692	2857262 **(65.9)**	1205092 **(42.2)**	30200 **(1.1)**
*r2d2^1^*/ *r2d2^1^*	+	3401343	1038959 **(30.5)**	273550 **(26.3)**	64192 **(6.2)**
**GERMLINE**					
*loqs-D*/+	–	14512820	10955066 **(75.5)**	2331242 **(21.3)**	2626560 **(24.0)**
*loqs-D*/+	+	14167951	10343590 **(73.0)**	65066 **(0.6)**	4955844 **(47.9)**
*loqs-D*/*loqs-D*	–	12343141	10107095 **(81.9)**	1288227 **(12.7)**	3342406 **(33.1)**
*loqs-D*/*loqs-D*	+	14963584	10910993 **(72.9)**	75443 **(0.7)**	4680467 **(42.9)**
*r2d2^1^*/+	–	13982564	10624983 **(76.0)**	1805164 **(17.0)**	3076304 **(29.0)**
*r2d2^1^*/+	+	5385640	4164837 **(77.3)**	25137 **(0.6)**	2012986 **(48.3)**
*r2d2^1^*/ *r2d2^1^*	–	5715078	3617578 **(63.3)**	548556 **(15.2)**	1125305 **(31.1)**
*r2d2^1^*/ *r2d2^1^*	+	6797261	5149557 **(75.8)**	15401 **(0.3)**	2244828 **(43.6)**

### Transposon-targeting piRNAs are unchanged if the endo-siRNA system is compromised

**Figure 1:**
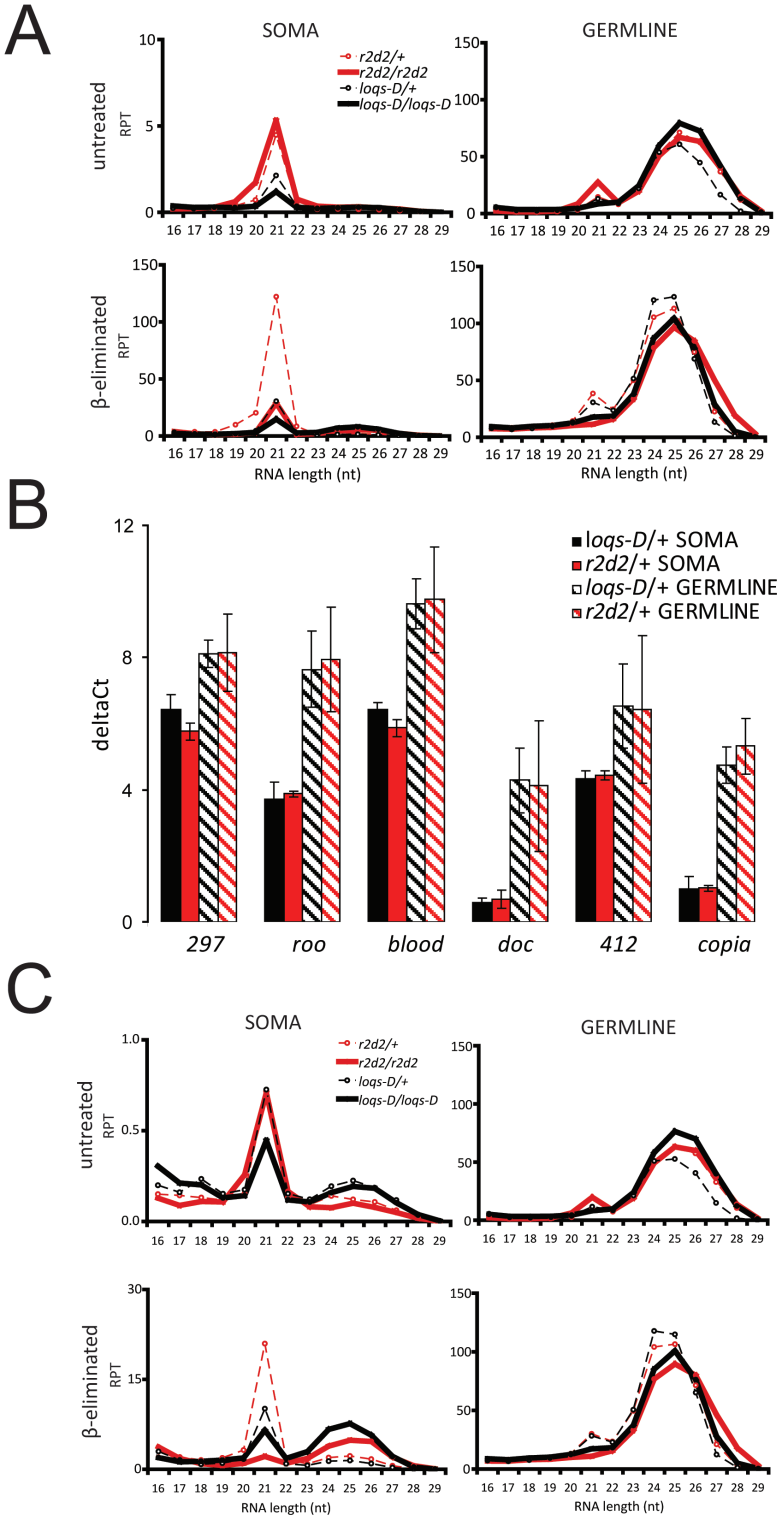
Length distribution of transposon-matching small RNAs identified in this study. A) Reads of each library originating from soma and germline were mapped to the reference containing a transposon sequence collection. Transposon matching small RNAs were analyzed for their size distribution and normalized to total genome matching reads. The normalized counts were expressed as reads per thousand (RPT). B) The steady state transcript levels of *297, TNFB, roo* and *blood* transposable elements were examined by qRT-PCR. RNA was isolated from three biological replicates of heterozygous *loqs-D* and *r2d2* mutants separated in somatic and germline tissue, respectively. The *doc, 412* and *copia* transposons were included for comparison. Ct-values for each transposon were normalized to the *rp49* control (delta Ct). Values are mean ± SD (n = 3). C) The length distribution of transposon matching small RNAs in *r2d2* and *loqs-D* mutants after exclusion of *roo*, *297*, *TNFB* and *blood* transposons.

Retrotransposons are transcriptionally very active in the germline and their efficient repression depends heavily on piRNAs. However, transposon-targeting endo-siRNAs are also robustly expressed in the germline and potentially compete with the piRNA system for antisense transcripts, which are required for dsRNA generation as well as for the ping-pong amplification cycle. We therefore asked whether impaired endo-siRNA biogenesis affects the germline piRNA profile. After mapping of the small RNAs to a collection of transposon sequences, the size distribution of the matching small RNA reads was profiled. We could distinguish peaks at 21 nt and at 24–27 nt reflecting the presence of endo-siRNAs and piRNAs, respectively (Figure 1 A). Consistent with the published literature, piRNAs were more abundant than endo-siRNAs in the germline and resistant to the β-elimination procedure [20], [21]. Neither their overall abundance nor the targeting of individual transposons by piRNAs changed in a manner that correlates with the state of the endo-siRNA pathway in our experiment, consistent with the results previously obtained for *dcr-2* mutants [7].

### Biogenesis of endo-siRNAs in the germline

A prevailing model for endo-siRNA biogenesis is that Loqs-PD acts predominantly during processing of dsRNA by Dcr-2, while the function of R2D2 is to ensure that the siRNAs are loaded into Ago2, rather than Ago1 [22]. However, exceptions to such a linear pathway exist [11], [12]. This model was derived from analysis of somatic or whole fly samples, but has not yet been validated in the germline where the very active transcription of transposons may impose differential requirements on endo-siRNA biogenesis. Our deep sequencing analysis revealed that in the germline, RNA samples obtained from homozygous *loqs-D* mutants contained a reduced number of 21-mer transposon-targeting endo-siRNAs both before and after β-elimination, consistent with a model where siRNA production by Dcr-2 is diminished without Loqs-PD (Figure 1 A, right panel). The abundance of transposon-targeting endo-siRNAs appeared increased in homozygous *r2d2* mutant animals; besides genetic background effects, this may indicate a competition between R2D2 and Loqs-PD for Dcr-2 binding. Most of the 21 nt long transposon-targeting endo-siRNAs derived from homozygous *r2d2* mutants were sensitive to β-elimination whereas the 21 nt size peak of RNA from the heterozygous controls remained. This has been attributed to loading of siRNAs into Ago1 in the absence of R2D2 [22] and is consistent with the results obtained in our initial survey of the somatic libraries (Figure S6 in File S1). Taken together, we could corroborate the existing model for the predominant endo-siRNA biogenesis pathway in the germline.

### Biogenesis of endo-siRNAs in the soma

In parallel to the germline analysis, we generated libraries from the somatic portions of flies to further differentiate the function of R2D2 and Loqs-PD during processing and loading. As for the ovary libraries, the reads were mapped to the transposon sequence collection with no mismatch allowed and their size distribution profiled. To allow for quantitative comparisons, the libraries were normalized to the total number of reads matching the *Drosophila* genome. A striking observation was that a large proportion of reads (0.6% to 14.5% of genome matching reads, 5.1% to 80.6% of transposons matching reads) could be attributed to only four transposable elements (*roo*, *297*, *TNFB* and *blood*) (Table 2). Diagrams that depict the normalized length distribution for each one of these transposable elements individually are included in Figure S7 in File S1). The amount of endo-siRNAs against *roo*, *297*, *TNFB* and *blood* in libraries was disproportionately high with respect to their steady state transcript levels (Figure 1 B). Compared to other transposons, they are either particularly efficiently targeted by the endo-siRNA system or strongly overrepresented in our deep sequencing libraries for unknown technical reasons. We did not detect any differences of potential biological relevance between this group (*roo*, *297*, *TNFB* and *blood*) and other transposons (see Figure S7 in File S1). Therefore, to allow for a more diversified representation of many distinct transposons, we present the remainder of the results in this manuscript with the reads matching those four mobile elements filtered out.

**Table 2:** The counts of *297, TNFB, roo* and *blood* matching small RNAs.

values normalized to transposon matching reads in the respective library														
untreated														
Soma		**r2d2/+**	**r2d2/r2d2**	**loqs-D/+**	**loqs-D/loqs-D**	Germline				**r2d2/+**	**r2d2/r2d2**		**loqs-D/+**	**loqs-D/loqs-D**
16–29 nt	297	6.01	12.45	9.93	6.03	16–29 nt		297		3.13	3.17		3.98	1.66
	TNFB	45.12	34.98	4.55	1.82			TNFB		1.18	2.97		0.1	0.01
	roo	19.59	33.27	32.06	37.36			roo		5.57	3.76		5.3	2.64
	blood	1.4	0.88	1.77	0.83			blood		2.76	1.6		1.69	0.81
	**sum**	**72.12**	**81.58**	**48.32**	**46.04**			**sum**		**12.65**	**11.5**		**11.07**	**5.13**
**β-eliminated**														
		**r2d2/+**	**r2d2/r2d2**	**loqs-D/+**	**loqs-D/loqs-D**					**r2d2/+**	**r2d2/r2d2**		**loqs-D/+**	**loqs-D/loqs-D**
	297	7.52	2.55	15.26	5.85			297		2.95	2.97		2.96	1.7
	TNFB	53.41	7.56	6.94	2.02			TNFB		1.54	0.06		0.06	0.01
	roo	17.92	5.8	29.99	15.73			roo		3.58	4.49		3.14	2.48
	blood	1.75	37.19	2.33	1.19			blood		2.05	1.76		1.46	0.89
	**sum**	**80.61**	**53.1**	**54.52**	**24.79**			**sum**		**10.13**	**9.28**		**7.63**	**5.08**
**untreated**														
Soma		**r2d2/+**	**r2d2/r2d2**	**loqs-D/+**	**loqs-D/loqs-D**		Germline			**r2d2/+**		**r2d2/r2d2**	**loqs-D/+**	**loqs-D/loqs-D**
16–29 nt	297	0.04	0.13	0.05	0.03		16–29 nt		297	0.91		0.99	0.95	0.55
	TNFB	0.34	0.37	0.02	0.01				TNFB	0.34		0.92	0.02	0
	roo	0.15	0.35	0.16	0.16				roo	1.61		1.17	1.27	0.87
	blood	0.01	0.01	0.01	0				blood	0.8		0.5	0.4	0.27
	**sum**	**0.54**	**0.86**	**0.25**	**0.2**				**sum**	**3.66**		**3.58**	**2.65**	**1.7**
**β-eliminated**														
		**r2d2/+**	**r2d2/r2d2**	**loqs-D/+**	**loqs-D/loqs-D**					**r2d2/+**		**r2d2/r2d2**	**loqs-D/+**	**loqs-D/loqs-D**
	297	1.35	0.16	0.77	0.32				297	1.43		1.3	1.42	0.73
	TNFB	9.57	0.47	0.35	0.11				TNFB	0.75		0.02	0.03	0
	roo	3.21	0.36	1.52	0.86				roo	1.73		1.96	1.51	1.06
	blood	0.31	2.3	0.12	0.06				blood	0.99		0.77	0.7	0.38
	**sum**	**14.45**	**3.29**	**2.77**	**1.35**				**sum**	**4.9**		**4.05**	**3.66**	**2.18**

Loss of Loqs-PD resulted in a 1.8-fold reduction of transposon-matching endo-siRNAs in libraries without β-elimination, consistent with the notion that its role is predominantly in siRNA production (Figure 1 C, left panel). While this was true for the analysis of all transposons in bulk, some individual exceptions to this rule exist. For example, the transposons *F-element*, *412* and *Doc* were only slightly affected by loss of Loqs-PD (Figure S8 in File S1). The overall reduction of transposon-targeting endo-siRNAs was also observed after β-elimination, in agreement with the notion that small RNAs must be produced before they can be loaded. As expected, this situation was different in the case of the *r2d2^1^* mutation: We observed no overall reduction of transposon matching endo-siRNAs between heterozygous and homozygous mutants before β-elimination (Figure 1 C, left panel) with only one major exception: The endo-siRNAs directed against *F-element* were strongly reduced in the absence of *r2d2* but only mildly affected by the absence of *loqs-D* (Figure S8 in File S1). After β-elimination, we observed a clear overall reduction of the 21-mer transposon matching siRNAs when R2D2 was absent. This is consistent with the published hypothesis that in the absence of R2D2, many endo-siRNAs are produced normally but loaded into Ago1 [22]. Yet, some siRNAs remained after treatment (Figure 1 C, left panel and Figure S6 in File S1) and thus appear to be correctly loaded in the absence of R2D2.

**Figure 2:**
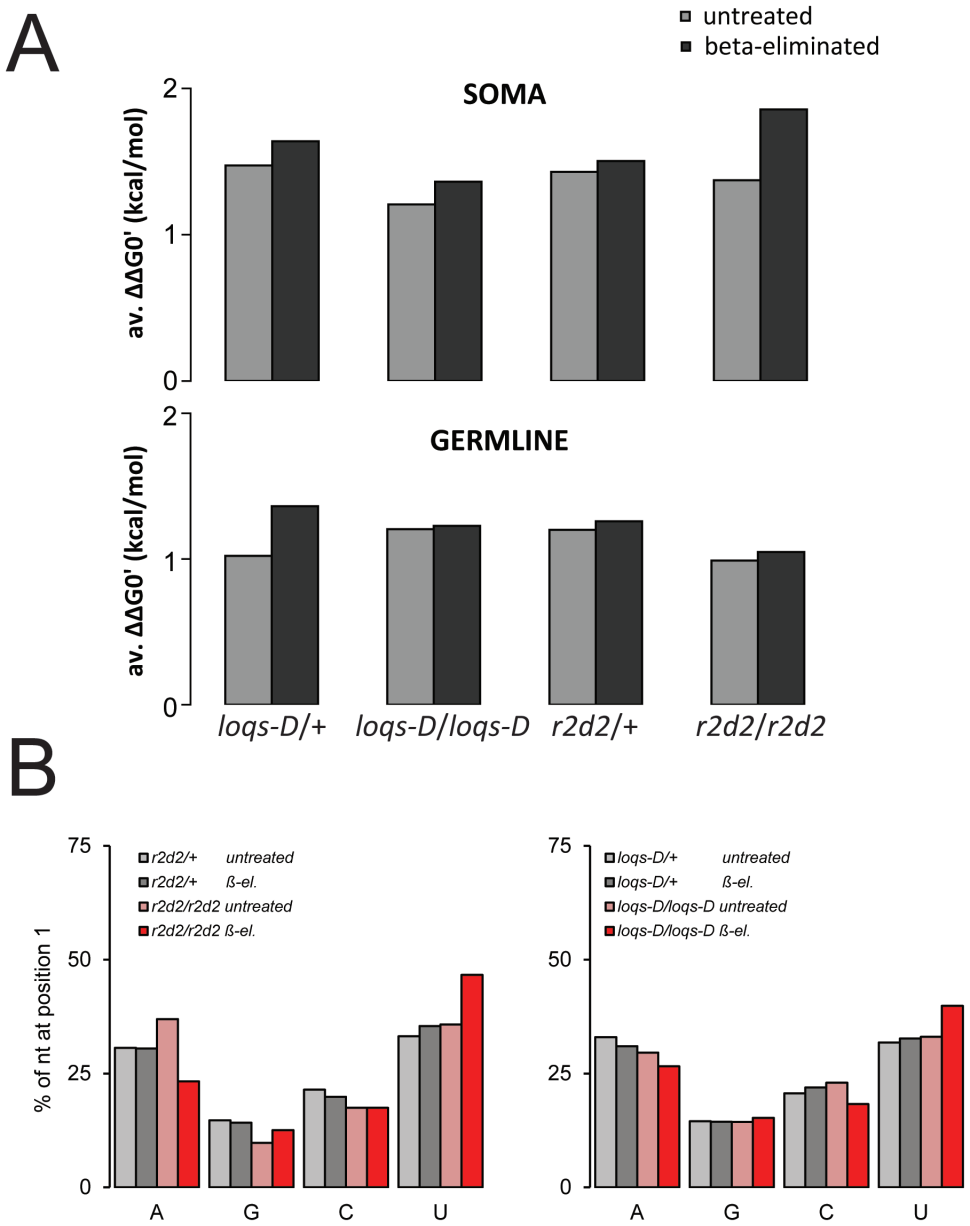
Analysis of strand asymmetry and 5’-nucleotide preference in deep sequencing data. A) The thermodynamic asymmetry was calculated for transposon mapping endo-siRNAs of the indicated genotypes. We calculated the difference in free energy of duplex formation at either end of the presumed siRNA precursor for each sequence read using the nearest neighbor method [51], then calculated the average difference (ΔΔG0'). A positive value indicates that on average the 5’ ends of the reads are less stably base paired than the opposite ends. B) The relative frequency for each nucleotide at the 5’-end is depicted as a function of genotype and RNA treatment.

To exclude that incomplete chemical oxidation/β-elimination may be a technical reason that could explain these *r2d2* independent siRNAs, we further verified that they not only differ from miRNAs in their susceptibility to the treatment (Figure S6 in File S1) but also show essential features of Ago2-loaded small RNAs. First, the *r2d2* independent siRNAs might represent double-stranded Dicer products that selectively escaped the derivatization. To rule out this possibility, we calculated the free energy of base pairing across the first 5 nt of each read at either end of the presumed duplex siRNA, then determined the difference between the 5’ and 3’ end of each presumed endo-siRNA precursor. We found a consistent excess of base-pairing stability of the 3’-end over the 5’-end of the small RNAs, indicating that they have been subject to a comparable extent of strand selection that follows the established rules (Figure 2 A). Second, the *r2d2* independent, β-elimination resistant reads may represent Ago1-loaded siRNAs (thus explaining the strand selectivity) that have escaped chemical treatment. In this case, we would expect that the 5’-nucleotide preference does not change with or without β-elimination as the same RNA pool would be sequenced in both cases. In the untreated RNA samples, loss of *r2d2* led to a small increase of transposon-matching siRNAs that begin with adenosine at their 5’-end, accompanied by a corresponding reduction of guanosine and cytosine in comparison to heterozygous controls. In contrast, upon β-elimination transposon-matching small RNAs from homozygous *r2d2^1^* animals showed a reduction of adenosine and a corresponding increase of uridine, clearly demonstrating that this small RNA population is distinct from the one that was sequenced without β-elimination (Figure 2 B). Finally, it is formally possible that in the absence of *r2d2* Ago1-loaded small RNAs become 2’-*O*-methyl modified by Hen-1. This is unlikely, however, since Ago1-loaded miRNAs remain sensitive to β-elimination in *r2d2^1^* mutant flies (Figure S6 in File S1 and [22]). Together with the observation that not all transposons are equally affected by the loss of *r2d2*, this is an indication that RLC action and strand selection can occur (though to a reduced extent) in the absence of *r2d2.*

**Figure 3:**
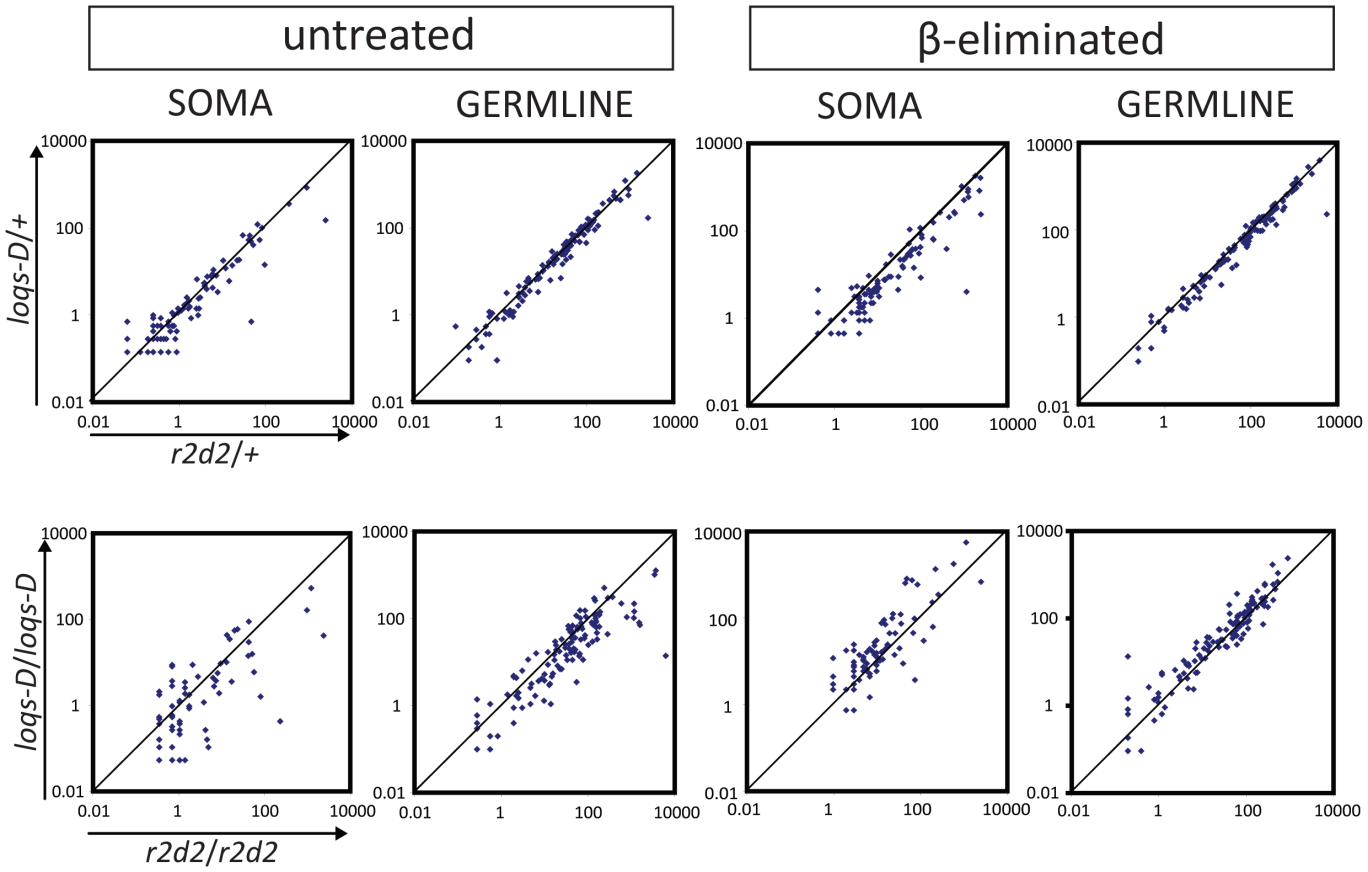
Changes in processing and loading of small RNAs matching to individual transposons in *r2d2* and *loqs-D* mutants. Transposon mapping endo-siRNA were normalized to total genome matching reads and expressed as reads per million (RPM). Each dot in the plot represents an individual transposon consensus sequence. The upper two panels compare of heterozygous *r2d2* and *loqs-D* mutants during processing (left) and loading (right) for soma and germline. The lower panels compare homozygous *r2d2* with homozygous *loqs-D* mutants. For example, a higher amount of endo-siRNAs in *r2d2* homozygous mutant than in *loqs-D* homozygous mutants means that these endo-siRNAs are *r2d2* independent but *loqs-D* dependent. They are thus situated below the diagonal, whereas transposons that require *loqs-D* but not *r2d2* will fall above the diagonal.

Is there any common principle that could explain why certain transposons differ from the bulk in their requirements for Loqs-PD and R2D2? This distinction is not based on their abundance since preference of Loqs-PD for biogenesis or R2D2 for Ago2-loading does not correlate with the absolute amount of small RNAs (Figure 3). Furthermore, when transposons were classified into long terminal repeats (LTRs), long interspersed elements (LINEs) and inverted repeats (IRs), we did not observe any consistent correlation that could explain R2D2 versus Loqs-PD preference (Figure S9 in File S1).

**Figure 4:**
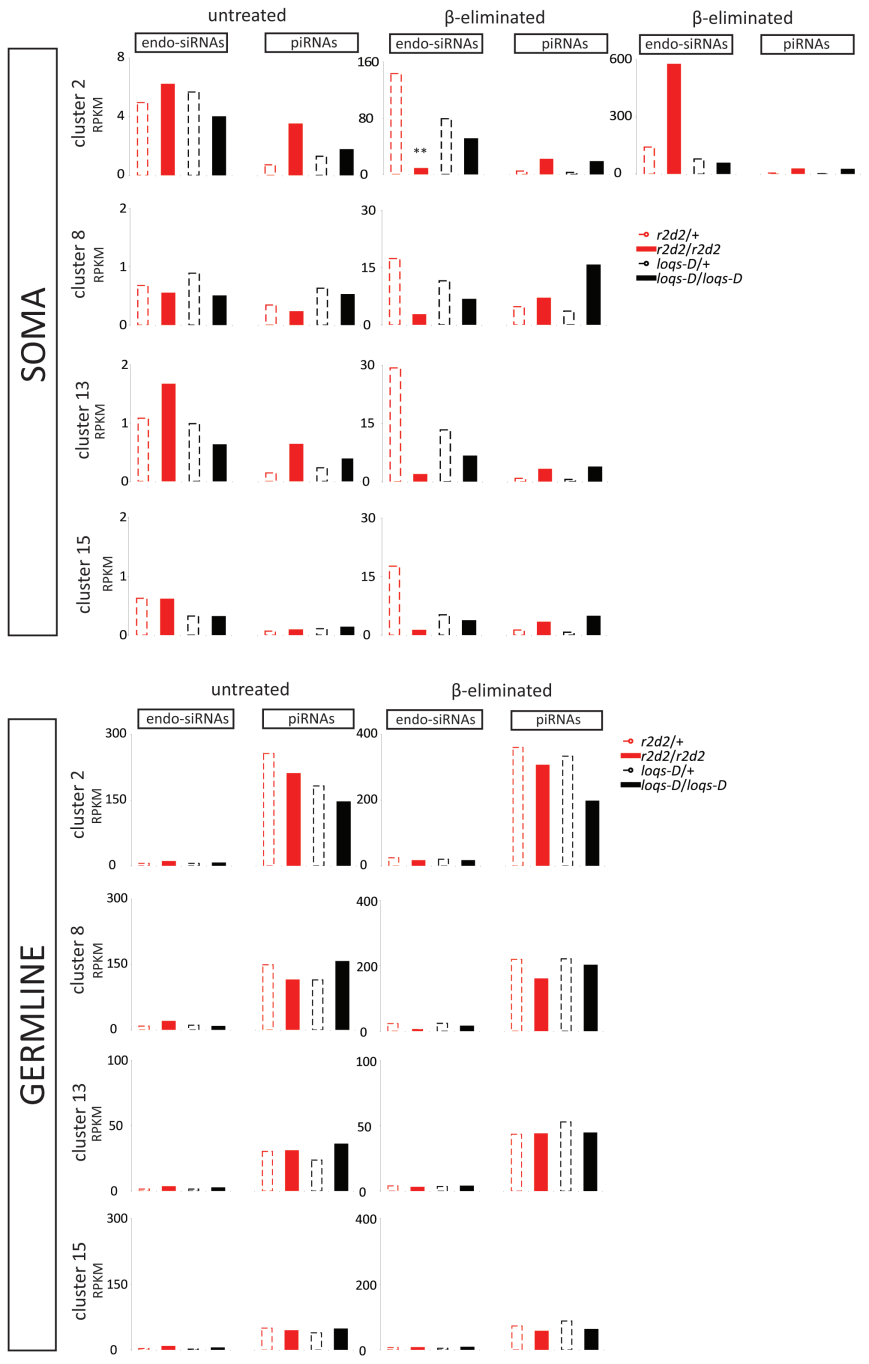
Analysis of endo-siRNA and piRNA origin with respect to a collection of transposon master control loci in the *Drosophila* genome. Fifteen transposon containing genomic regions were reported as master regulators of transposon activity [23]. The reads from each library were separated in endo-siRNAs (21 nt) and piRNAs (24-27 nt) by their length, then mapped allowing only those reads that matched uniquely among these clusters to be retained. The counts were normalized to cluster length as well as to total genome matching reads (reads per kilobase per million mapped reads, RPKM). Cluster 2 (chromosome X; 20A), 8 (chromosome X; 20 A-B, also known as *flamenco*), 13 (chromosome 3LHet) and 15 (chromosome 3LHet) are shown. In the soma cluster 2 showed an excessive amount of a unique sequence at a particular location in the homozygous mutant *r2d2* sample after β-elimination. This likely represents a technical artifact and was therefore omitted. We specifically label the results after exclusion of the special sequence with **.

As we could find no defining feature intrinsic to the transposons, we analyzed whether a particular genomic origin of the reads could explain R2D2 versus Loqs-PD dependence. To this end, we mapped deep sequencing reads to a collection of transposon containing genomic clusters [23], retaining only those reads that mapped uniquely among these clusters for analysis. One cluster on chromosome X (referred to as cluster 2, also known as 20A) generated a particularly high number of endo-siRNAs, which we detected both before and after β-elimination. Presumably, this reflects active bi-directional transcription of this cluster in somatic cells. After β-elimination we noticed an increased endo-siRNA amount in soma in the absence of *r2d2* in contrast to other clusters. This difference was due to a unique sequence with 23275 counts at a single location. We consider this sequence to be a technical artifact (e.g. particularly high ligation efficiency) and removed it from the analyzed data set (Figure 4, marked with **). This resulted in a consistent decrease of β-elimination resistant endo-siRNAs upon mutation of *r2d2* for all clusters. All in all, we saw no correlation between the site of genomic origin and dependence on Loqs-PD and R2D2 during either processing of the dsRNA precursor or loading of siRNA into Ago2.

### Endo-siRNAs contribute to transposon repression in soma and germline

**Figure 5:**
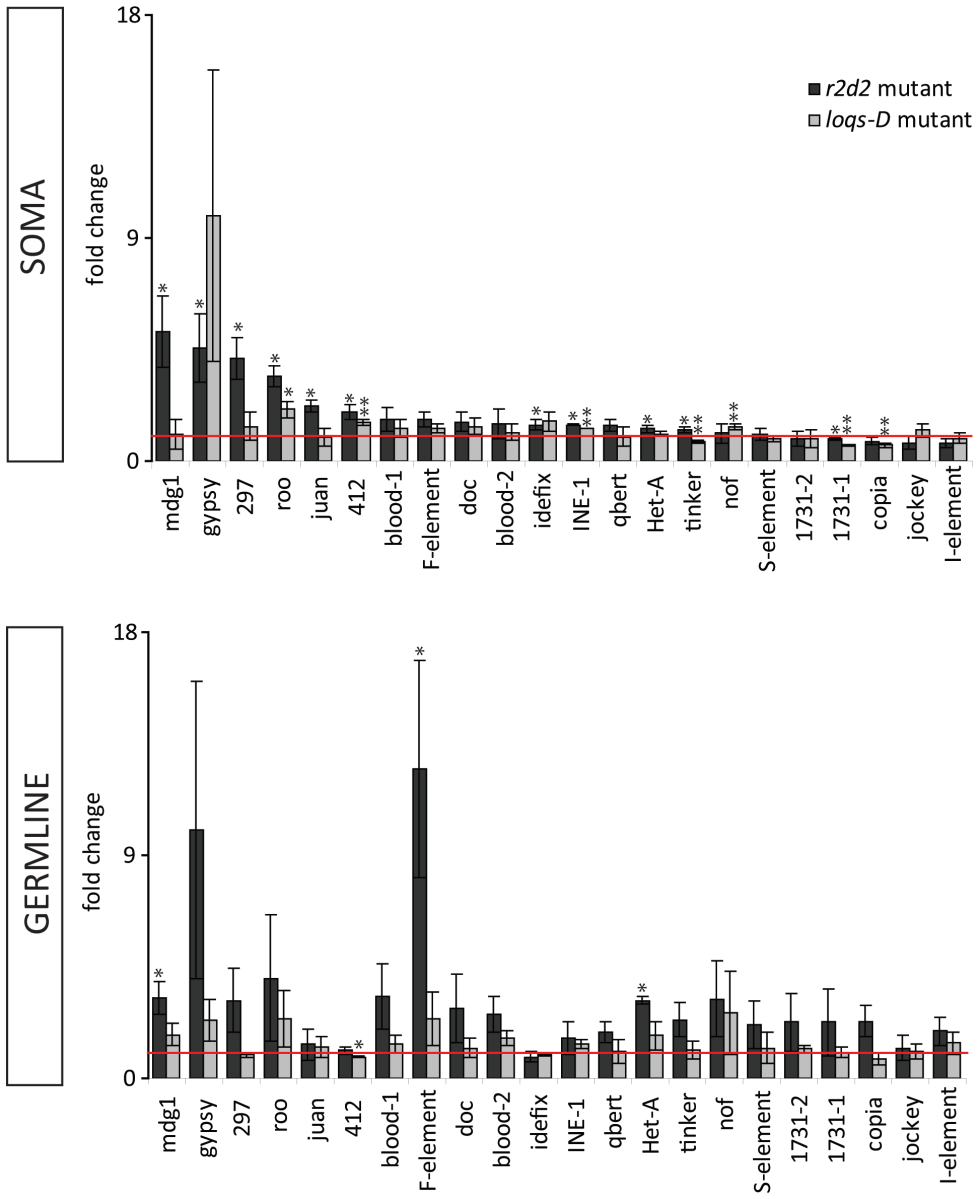
Analysis of steady state level of transposons by qRT-PCR. RNA was isolated from heterozygous and homozygous *r2d2* and *loqs-D* mutants. DNA was digested with DNase I, the RNA was reverse transcribed and used for transposon profiling by qRT-PCR. Each transposon was normalized to the average of *rp49* and *gapdh* controls and depicted as the fold change of homozygous to heterozygous mutant in soma and germline, respectively (p<0.05(*), p<0.009(**) student’s T-test, n = 3).

Do the differences in endo-siRNA abundance in response to Loqs-PD or R2D2 deficiency lead to changes in the steady state level of transposons? We analyzed RNA isolated from soma and ovaries of heterozygous and homozygous flies, then determined the transcript levels of 22 distinct transposons by quantitative RT-PCR. The difference between homozygous and heterozygous mutants is presented as fold change in expression (Figure 5). In the somatic sample, loss of R2D2 resulted in de-repression of the transposons *mdg1*, *gypsy*, *297*, *roo*, *juan*, *idefix* and *412* (t-test, p≤0,05). Loss of Loqs-PD, in contrast, only resulted in de-repression of *412*, *roo*, *INE-1* and *nof* (t-test, p≤0,05). Apparently, the redirection of endo-siRNAs into Ago1 in the absence of R2D2 represents a more severe loss of function than the reduced endo-siRNA biogenesis upon loss of Loqs-PD. In the germline, a high abundance of piRNAs and the severe phenotype of piRNA pathway mutations suggest a predominance of the piRNA system in transposon repression. Nonetheless, we detected significant changes for *mdg1*, *het-A* and *F-element* upon loss of R2D2 in ovarian RNA samples. *Mdg1* is expressed predominantly in the somatic follicle cells [24] present in our ovary preparation but *HeT-A* as well as the *F-element* are considered mostly germline expressed [21], [25]. Furthermore, the *412* element with preferential expression in the follicle cells is unchanged in *r2d2^1^* mutants and slightly hyper-repressed in *loqs-D* mutant ovaries. Taken together, we could demonstrate that a certain extent of transposon de-repression can be observed if the endo-siRNA pathway is impaired in the germline. We examined whether the changes in steady-state transposon mRNA levels correlate with the effects of *r2d2^1^* or *loqs-D* mutants on endo-siRNA abundance. The corresponding scatter plots comparing the fold change of mRNA levels against the fold change in siRNA levels from homozygous versus heterozygous mutants indicate that in both soma and germline, a reduction of transposon targeting endo-siRNAs can occur without necessarily affecting the corresponding steady-state mRNA levels (Figure S10 in File S1).

### Occurrence of somatic pilRNAs

**Figure 6:**
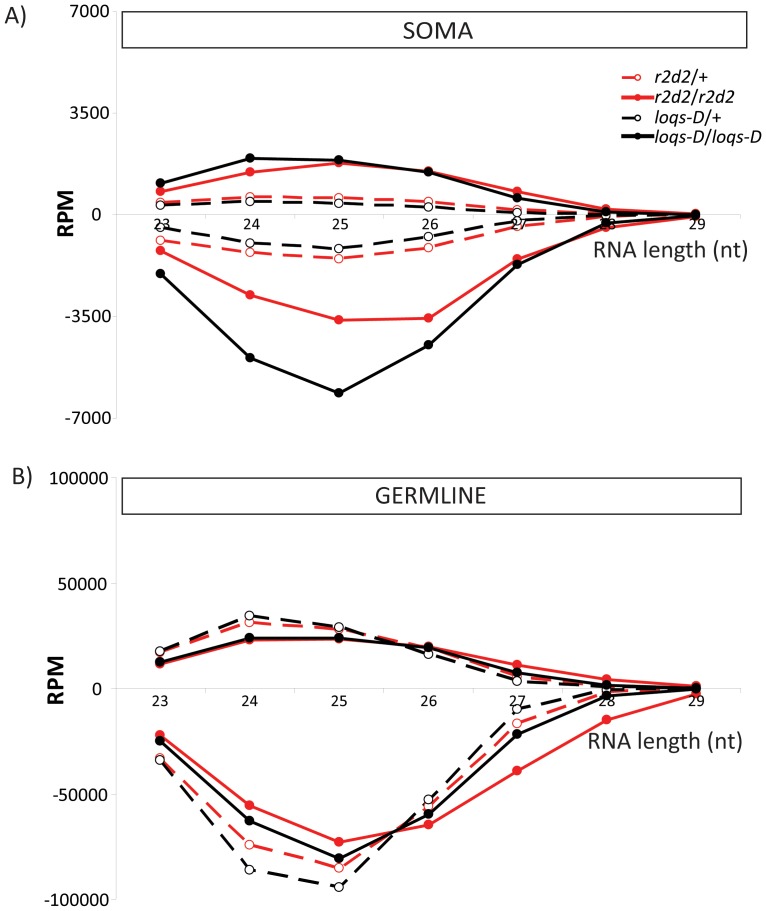
Orientation bias for pilRNAs in soma and piRNAs in germline. Small RNA libraries generated with β-eliminated RNA samples were mapped to the transposon sequence collection. The RPM for sense (+) and antisense (–) transposon matching small RNAs for 23 nt to 29 nt were depicted for soma (A) and germline (B) to demonstrate the orientation bias. Note that the apparent increase of somatic pilRNAs is due to the removal of certain miRNAs and endo-siRNAs in homozygous mutants, which are either less efficiently produced and/or mis-directed into Ago1. Upon β-elimination, these RNAs no longer contribute to the sequenced pool, hence other RNA classes appear to be more abundant. We did not observe this increase if untreated libraries were analyzed.

Upon mapping of deep sequencing reads to the transposon consensus sequences, somatic small RNA libraries also indicated the presence of RNAs in the size range of 24 to 27 nt. Such somatic piRNA-sized species have been previously described in the heads of *ago2^414^* mutant flies [4] as well as in mouse and rhesus macaque samples [26]. They are referred to as piRNA-like small RNAs (pilRNAs). If these small RNA species are loaded into either Ago2 or a Piwi-family effector protein they should be 3'-end modified and resistant to β-elimination. Indeed, we found that transposon targeting, 24 to 27 nt long RNAs were enriched in the β-eliminated small RNA libraries (Figure 1 C). Like germline piRNAs, they showed a bias towards antisense orientation (Figure 6), which argues against Dicer-dependent processing of a double-stranded precursor. We generated sequence logos of 24–27 nt long sense and antisense matching reads separately. A strong preference for a 5'-U in the antisense matching reads could be seen whereas sense-matching reads in this size-range showed a preference for U at the first position and/or an A at 10th position. These features are clearly visible in all samples after β-elimination and indicative of biogenesis via the ping-pong mechanism [21], [23], [27]. In the case of germline piRNAs, the preference for A at position 10 of sense piRNAs can also be seen in the untreated sample, while the somatic samples likely contain transposon mRNA degradation products that mask this feature in the untreated libraries. We further confirmed the ping-pong signature by determining the sequence overlap between sense- and antisense-matching reads of 24–27 nt length. A pronounced peak at a 10 nt overlap was present in all cases for the beta-eliminated samples, while the non-treated somatic samples did not show a clearly discernible peak at 10 nt of overlap (Figure S11 in File S1).

The biogenesis of piRNAs is based on Piwi-family proteins with Ago3 as the predominant carrier of sense piRNAs while Piwi and Aub bind antisense piRNAs. If somatic piRNAs exist and are produced via the ping-pong mechanism, then Piwi-family proteins should be expressed in the soma as well. We tested this by RT-PCR and found expression levels close to background, whereas the corresponding transcripts were readily detectable in the germline (Figure S12 in File S1). This could either indicate that most somatic cells express very low levels of Piwi-family genes, or that a small subset of the somatic cells in adult flies is proficient for the piRNA pathway. In the first scenario, a homogeneous somatic cell population should show an amount of piRNA-sized transposon-matching reads comparable to our somatic fly libraries. We analyzed published small RNA sequencing libraries from the somatic S2 cell line of embryonic origin [11] but found no indication that pilRNA reads were present. The most likely explanation for the origin of somatic piRNAs is therefore that a small subset of cells with an active piRNA pathway including the ping-pong mechanism exists in the soma of flies.

## Discussion

### Lack of a dsRBP protein co-factors leads to defects in endo-siRNA biogenesis

The discovery of transposon-targeting endo-siRNAs was accompanied by experiments that elucidated whether their biogenesis and function depends on the known siRNA-pathway components Dcr-2 and Ago2. If one of these factors is missing, endo-siRNAs are reduced in abundance and a small extent of transposon de-repression can be measured in soma as well as ovaries. We generated deep sequencing libraries to analyze the relative contribution of the dsRBD protein cofactors Loqs-PD and R2D2 during endo-siRNA biogenesis in soma and germline. These libraries have allowed us to verify that impaired endo-siRNA biogenesis does not influence the piRNA system in soma or germline. The sterile phenotype of many piRNA pathway mutations [21], [28], [29] contrasts a comparatively mild impairment of e.g. *dcr-2* or *ago2* null mutations [30], [31]. Clearly, the piRNA system has more impact on transposon activity than the endo-siRNA system. It should be noted, however, that many of the genetic experiments measured female fertility. The piRNA system also exists in the male germline, but the male sterile phenotype of the siRNA-specific factor *blanks* [32] indicates that endo-siRNAs may play a more pronounced role in transposon defense in the male germline.

The amount of piRNAs present in ovaries is indeed tremendous compared with the abundance of endo-siRNAs. In this context it is surprising that impaired endo-siRNA biogenesis resulted in a measurable impact on the steady state transcript levels for a small number of transposons (see Figures 1 and 5). This indicates their biological significance despite the comparatively lower abundance. In the soma, the majority of transposons in *loqs-D* and *r2d2^1^* mutants was unchanged (Figure 5), the effects of removing either dsRBP protein cofactor is therefore weaker than the one described for the *dcr2^L811fsX^* and *ago2^414^* null mutations [33]. To exclude the possibility that part of the transposon transcripts remain undetected because of sequence variation within primer binding sites, we also tested an alternative primer pair for the transposons blood, mdg1, 297 and 1731. Comparable results were obtained (data not shown), indicating that our analysis is likely representative of the major transcript pool. It further appears that endo-siRNAs are usually generated in excess, as the reduction observed in *loqs-D* mutants did not result in a clear increase of steady state transposon mRNA levels. In contrast, the redirection of endo-siRNAs into Ago1 in the *r2d2^1^* mutant [22] resulted in a slight de-repression for some transposons. This is consistent with the observation that Ago1 has a lower catalytic rate and dissociates inefficiently from the cleavage products [18].

### Overlapping function of Loqs-PD and R2D2

The analysis of small RNAs involved in protection against transposable elements demonstrated that Loqs-PD acts predominantly during processing of dsRNA by Dcr-2, while the function of R2D2 is to ensure that siRNAs are loaded into Ago2. This model can explain many of the molecular features and biogenesis requirements that were identified for transposon-targeting and other endogenous siRNAs. However, *loqs* and *r2d2* double-mutant flies show a more severe endo-siRNA deficient phenotype [12], indicating that the processes of production and loading/stabilization may be mechanistically linked and/or that a certain extent of redundancy between Loqs-PD and R2D2 exists. We now provide additional support consistent with the hypothesis that Loqs-PD and R2D2 can have overlapping functions. First, a considerable amount of endo-siRNAs remained in the *loqs-D* mutant, indicating that Loqs-PD is not required for dicing of all endo-siRNA precursors. Basal activity of Dcr-2 in the absence of any dsRBD protein cofactor may certainly account for part of these siRNAs; however, Carthew and colleagues demonstrated that extracts derived from *loqs, r2d2* double mutant embryos were dicing a 200 nt long dsRNA substrate less efficiently *in vitro* than extracts from either single mutant alone [12]. Second, in the absence of *r2d2* our libraries showed that some transposon-targeting endo-siRNAs remain Ago2-loaded. A recent publication demonstrated that an important function of R2D2 is to assist in the formation of cytoplasmic D2-bodies by interacting with Dcr-2 and RNA. While the formation of D2-bodies is a prerequisite to prevent large-scale mis-loading of siRNAs into Ago1, a low amount of siRNA-loading into Ago2 occurred even in the absence of R2D2 [34]. This is fully consistent with our observation of β-elimination resistant, transposon-targeting siRNAs that have undergone strand-selection in *r2d2^1^* mutant flies. These siRNAs show a moderate preference to begin with a 5’-uridine (Figure 2 B), a feature that we had previously observed in sequencing data obtained from mutant fly heads [11]. As *loqs-D* homozygous mutant flies also show an increase of transposon-targeting endo-siRNAs with a 5’-uridine upon β-elimination (though of lower magnitude), a possible explanation is that a small amount of mis-targeting already occurs in the absence of Loqs-PD despite the presence of R2D2. A direct comparison of the sensitivity to β-elimination with miRNAs in heterozygous and homozygous *loqs-D* mutants also indicates that *loqs-D* is required to achieve the full extent of Ago2-loading (Figure S6 in File S1).

The role of R2D2 for the production of endo-siRNAs differed when comparing soma with germline (Figures 1 and 5). In the soma, processing was mostly independent of *r2d2* while the absence of *r2d2* in germline resulted even in an increased production of endo-siRNAs. This was not caused by a small group of transposons that respond atypically but rather was visible for most TEs which generated endo-siRNAs in germline (Figure S9 in File S1). Thus, R2D2 appears to reduce the yield of dsRNA processing in the germline, indicating a potential competition between R2D2 and Loqs-PD for association with Dcr-2. Due to the strong expression of transposons in the germline, endo-siRNA biogenesis presumably occurs at a higher rate and such a competitive phenomenon may therefore be easier to observe in germline than in the soma. Both R2D2 and Loqs-PD were shown to interact with an equivalent position on Dcr-2, the helicase domain [11]. Consistent with a competition for Dcr-2 association, depletion of R2D2 increased the efficiency of endo-siRNA mediated silencing in *Drosophila* cell culture [9]. We note that the human dsRBD proteins TRBP and PACT were reported to have antagonistic effects on Dicer as TRBP stimulates miRNA dicing and stabilizes Dicer while PACT inhibits miRNA processing [35]–[37].

Why do certain transposons differ from the bulk in their preference for Loqs-PD and R2D2? They are not distinguished based on the abundance of corresponding siRNAs (Figure 3). Furthermore, we could not find a correlation between the differential requirement for Loqs-PD or R2D2 and specific transposon classes or their presence in a particular master control locus (Figure 4 and Figure S9 in File S1). Tissue-specific differences in transposon expression may nonetheless have masked a potential correlation between transcriptional activity and a requirement for Loqs-PD or R2D2. We isolated RNA from complex tissues (head + thorax vs. ovaries) and our data does not allow us to distinguish if the expression of a given transposon is strong but restricted to a fraction of the cells, or moderate and ubiquitous.

### Confirmation and characterization of somatic piRNA-like RNAs

The Piwi-interacting RNA pathway preserves the integrity of the genome in the germline, guarding it against the activity of mobile elements. We could further detect piRNA-like RNAs (pilRNAs) with 23 to 27 nt length in soma matching transposons but present in significantly smaller quantity than germline piRNAs or somatic endo-siRNAs (Figure 1). These small RNAs were 2’-*O*-methyl modified as demonstrated by their enrichment after β-elimination. Evidence for the occurrence of somatic pilRNAs has been scarce in the literature. A first description of *Drosophila* pilRNAs was from libraries of *ago2* mutant heads, including the characteristic 2’-*O*-methyl group at their 3’-end [4]. Furthermore, pilRNAs were observed in multiple somatic tissues of mouse and rhesus macaque as well as human natural killer cells (NK) [26], [38], [39]. The majority of germline piRNAs tend to be antisense to transposons [23] and we saw the same orientation bias in the soma (Figure 6). The confirmation of a ping-pong signature in the sequences of somatic pilRNAs indicates that a full piRNA pathway is active in the soma as well. This is in contrast to ovarian follicle cells, which only harbor primary piRNAs. The full piRNA pathway in germ cells requires presence of all three Piwi-family proteins: Piwi, Aub and Ago3 [23], [27]. Somatic piRNA-like RNAs are therefore expected to require the same set of proteins; yet, expression of these factors in somatic cells (other than Piwi in the follicular sheath) has not been well documented in *Drosophila*. Our qRT-PCR analysis also did not yield convincing evidence of robust transcription within our complex tissue samples. Gene expression studies published by the ModEncode consortium nonetheless reveal low-level expression of *piwi*, *aub* and *ago3* during all life stages. Furthermore, certain cell lines derived from imaginal discs show intermediate expression levels. A straightforward interpretation is that a small subset of somatic cells expresses sufficient amounts of the Piwi-familiy proteins to sustain or re-initiate production of primary and ping-pong piRNAs. Consistently, *in situ* hybridization in several adult macaque tissues indicated that pilRNA expression is restricted to specific cell types [26]. Furthermore, our analysis of transposon-targeting small RNAs from the somatic Schneider cell line did not reveal any pilRNAs, providing evidence that cells completely lacking the piRNA pathway exist. Expression of a human Piwi homolog, Hiwi, has been detected in CD34^+^ hematopoietic progenitor cells [40] and the planarian homolog Smedwi-2 is present in adult stem cells [41]. In *Drosophila*, ectopic expression of germline genes, including piRNA pathway factors, induces the formation of malignant brain tumors [42]. Finally, the recent observation of transposon mobility during the establishment of the adult *Drosophila* brain along with expression evidence for *aub* and *ago3* even suggests that there may be a physiologic role for transposon repression and de-repression via the piRNA system in somatic cells [43]. It is thus tempting to speculate that the pilRNAs originate in e.g. somatic stem cells and that these cells express a fully functional piRNA pathway. Several assays have reported somatic phenotypes for *piwi* mutations [44]–[46], in particular associated with heterochromatic silencing and dosage compensation. Given the capacity of piRNAs to regulate transcription [47], [48] their role in stem cells might be to instruct specific chromatin structures, which are then maintained in the descendent differentiated cells.

## Materials and Methods

### Backcrossing of *loqs^ko^* and *r2d2* mutants

Transposons are a major source of genome variability and their activity and genomic distribution may differ between fly strains. To facilitate our comparative analysis of the *loqs^ko^* [14] and *r2d2* mutants [49], which derived from distinct genetic backgrounds, we performed one round of backcrossing for both mutations using *w^1118^* stock. Details are provided in the File S1.

### RNA isolation and qRT-PCR

Dissected ovaries or the head & thorax portion were ground in Trizol (Invitrogen; Carlsbad/CA, USA) using a micro-pistil. RNA was then extracted and precipitated according to the manufacturer’s instructions. For qRT-PCR analysis, the background genomic DNA was removed by digestion with DNaseI (Fermentas, St. Leon-Rot, Germany) followed by digestion with proteinase K (Fermentas, St. Leon-Rot, Germany). Reverse transcription was primed with random hexamers, then qRT-PCR was performed with the Dynamo Sybr Green System (Biozym, Hessisch Oldendorf, Germany) using the primers detailed in the section.

### β-elimination of total RNA

40 µg total RNA dissolved in 40.5 µl H_2_O and incubated with 12 µl 5x borate buffer (148 mM borax, 148 mM boric acid pH 8.6, 1% SDS) and 7.5 µl NaIO_4_ (200 mM feshly dissolved in H_2_O) for 10 min at RT. The oxidation was quenched by addition of 6 µl 100% glycerol (10 min, RT). Elimination of the oxidized last nucleotide was performed by elevating the pH with 2M NaOH (5–7 µl, to reach pH = 12). After 90 min at 45°C the sample was transferred to a Mini quick spin oligo column (Roche Diagnostics; Mannheim, Germany) for purification and centrifuged (12 000 x g, 2 min). 20 µg glycogen were added and RNA was precipitated with three volumes of 100% ethanol (12 000 g, 15 min). The RNA pellet was washed three times with 70% ethanol (last step 4°C, o/n) and dissolved in 20 µl 2x formamide gel loading buffer. A small aliquot of the samples were analyzed on a 15% acrylamide-urea and stained with sybr gold to verify complete β-elimination and RNA quality.

### Deep sequencing and data analysis

Small RNAs were enriched by size-selection on 20% acrylamide-urea gels, the size range of 19–29 nt was cut out and eluted from the gel slice. Linker ligation, library preparation and deep sequencing on the Illumina GAIIx platform was performed as previously described [11]. The sequences were mapped onto the target sequences using BOWTIE [50] with the option –n0 to force selection of only perfectly matching sequences. Pre-processing of sequences and analysis of the BOWTIE output files were done using PERL scripts (available upon request). For the mapping to individual clusters presented in Fig.4, we used the coordinates published by Brennecke et al. [23] to download the corresponding DNA seuqences from Flybase. These were assembled into a multiline FASTA file which we the used to build a reference index. Small RNAs were mapped to this collection of piRNA clusters without mismatch and we retained only those reads that mapped uniquely among the clusters (comparable results are obtained if this filter is omitted). To account for different cluster sizes (mapping efficiency is a function of sequence length), we normalized the reads not only to the sequencing depth of each library but also to the size of each cluster. We chose the RPKM nomenclature (**r**eads **p**er **k**ilobase of target sequence and **m**illion genome-matching sequences in library) in analogy to a commonly used approach for RNAseq.

The sequences obtained in this study were submitted to the NCBI GEO database under the accession number GSE45290.

## Supporting Information

File S1 Figure S1: Backcrossing scheme of *loqs^ko^* mutant in *w^1118^* genetic background. Figure S2: Fly stock mapping of *loqs^ko^* mutants. Figure S3: Backcrossing schema of *r2d2^1^* mutant in *w^1118^* genetic background. Figure S4: Fly stock mapping of *r2d2* mutants. Figure S5: Verification of β-elimination efficiency. Figure S6: Comparison of read counts in untreated and β-eliminated deep sequencing libraries (somatic RNA samples). Figure S7: Read length distribution of *roo*, *TNFB*, *blood* and *roo* transposon mapping small RNAs in *r2d2* and *loqs-D* mutants.. Figure S8: The length distribution of 412, F-element, doc transposon mapping small RNAs in *r2d2* and *loqs-D* mutants. Figure S9: Analysis of endo-siRNAs classified in LTRs, LINEs and IRs transposons in *r2d2* and *loqs-D* mutants. Figure S10: Comparison of endo-siRNA abundance changes with changes in steady-state levels of transposons. Figure S11: Analysis of ping-pong signature of pilRNAs and piRNAs. Figure S12: Transcript levels of *ago3*, *aub* and *piwi*.Table S1: Primer sequences.
(DOCX)
