# Supplementary material for: Transposon Defense by Endo-siRNAs, piRNAs and Somatic pilRNAs in Drosophila: Contributions of Loqs-PD and R2D2
Source: PLoS One. 2014 Jan 13;9(1):e84994. doi: 10.1371/journal.pone.0084994 (PMC3890300; doi:10.1371/journal.pone.0084994)
Supplement: File S1 — Figure S1: Backcrossing scheme of loqsko mutant in w1118 genetic background. Figure S2: Fly stock mapping of loqsko mutants. Figure S3: Backcrossing schema of r2d21 mutant in w1118 genetic background. Figure S4: Fly stock mapping of r2d2 mutants. Figure S5: Verification of β-elimination efficiency. Figure S6: Comparison of read counts in untreated and β-eliminated deep sequencing libraries (somatic RNA samples). Figure S7: Read length distribution of roo, TNFB, blood and roo transposon mapping small RNAs in r2d2 and loqs-D mutants.. Figure S8: The length distribution of 412, F-element, doc transposon mapping small RNAs in r2d2 and loqs-D mutants. Figure S9: Analysis of endo-siRNAs classified in LTRs, LINEs and IRs transposons in r2d2 and loqs-D mutants. Figure S10: Comparison of endo-siRNA abundance changes with changes in steady-state levels of transposons. Figure S11: Analysis of ping-pong signature of pilRNAs and piRNAs. Figure S12: Transcript levels of ago3, aub and piwi.Table S1: Primer sequences. (DOCX) [file pone.0084994.s001.docx]

Supplementary Material

Supplementary Material and Methods

#### **Backcrossing scheme for the** *loqs^ko^* **mutant**

The null allele *loqs^ko^* was generated through ends-out homologous recombination [[1](#_ENREF_1)]. As a result, the entire *loqs* open reading frame (ORF) was replaced with a *mini-white* transgene which serves as a marker gene. Park and colleagues described that the early development occurred normally while the viability dropped precipitously at the transition from pupa to adult stadium. 90% of the mutant flies died during eclosion and the remaining flies died after emerging. The *loqs* isoform Loqs-PB was shown to be essential to avoid defects in embryonic development and GSC maintenance [[2](#_ENREF_2)]. As our interest is centered on Loqs-PD, we used a fly strain that also carried a Loqs-PB transgene on the 3^rd^ chromosome. Virgins of a *loqs^ko^* mutant with restored Loqs-PB function were crossed with wild type *w^1118^* males (P, suppl. Fig. 1). After the selection against *Cy* and *Sb*, F1 offspring was further crossed with *Kr/Cyo; D/TM6C, Sb, Tb* double balancer males, to obtain offspring with balanced 2nd (*CyO*) and 3rd (*TM6, Tb, Sb*) autosomes. The following sibling mating (1 female + 1 male) allowed generation of stable balanced stocks producing homozygous and heterozygous *loqs^ko^* mutants rescued with a Loqs-PB cDNA construct.

The sibling mating resulted in a number of fly lines which were analyzed by genomic PCR with an appropriate primer pair within the first two exons of the *loqs* locus. The *loqs-PB* transgene consisted of cDNA lacking intronic sequences, therefore PCR products from the wild type *loqs* locus and *loqs-PB* cDNA could be distinguished by the size (320 nt versus 247 nt, respectively). All *loqs^ko^* fly stocks (lane 1 to 9 except lane 5) showed deletion of both *loqs* alleles according to the missing product at 320 nt, while Loqs-PB was present according to the 247 nt product (suppl. Fig. 2). As a control, the heterozygous *loqs* mutant, employed in the first crossing step (suppl. Fig. 1, P), was verified and confirmed to the appropriate genotype (suppl. Fig. 2, lane 10). With regard to the following analysis two fly stocks were selected (lane 3 and 7) and their corresponding heterozygous mutants were further analyzed to complete the characterization of the genotype. They were recognized due to the curly wing marker (*Cy*). PCR products demonstrated one wild type copy of the *loqs* locus (320 nt) as well as *loqs-PB* cDNA (247 nt) as expected (suppl. Fig. 2).

#### **Backcrossing scheme fo**r the *r2d2^1^* **mutant**

The *r2d2^1^* deletion flies were generated by imprecise excision of a P-element inserted near the *r2d2* locus [[3](#_ENREF_3)]. This resulted in a 4.9 kb deletion, which removed the entire *r2d2* ORF as well as 1 kb of upstream and 3 kb of downstream sequences, resulting in a null mutant allele. The deletion of the 4.9 kb region could be rescued by an intact *r2d2* gene [[3](#_ENREF_3)].

To obtain *r2d2* mutant flies with a genomic background more similar to the *loqs^ko^* mutant described above, virgins of the *r2d2* mutant were crossed with wild type *w^1118^* males (P, suppl. Fig. 3). F1 offspring was further crossed with *Kr/Cyo; D/TM6C, Sb, Tb* double balancer males to obtain offspring with balanced 2nd (*CyO*) and 3rd (*TM6, Tb, Sb*) autosomes. The deletion of *r2d2* is not recognizable by a marker gene. The second crossing step produced heterozygous mutants where the 2^nd^ chromosome was balanced over *CyO* and contained either wild type *r2d2* or deleted *r2d2*. The following sibling mating (1 female + 1 male fly) generated distinct genetic compositions, which could not be distinguished by visible markers (suppl. Fig. 3). Genomic PCR was the method of choice for recognition of deleted *r2d2* by using a combination of primer comprising *herp* (CG14536, upstream of *r2d2*), *r2d2* and *cdc14* (CG7134, downstream of *r2d2*) in a collection of derived fly stocks (suppl. Fig. 4, lane 3 to 9). The PCR conditions used for *herp* and *cdc14* primer did not allow amplification of the 6.3 kb PCR product in the wild-type sample. From mutants with straight wings (possibly homozygous genotype), lane 4 and 6 were selected as the flies were devoid of a wt *r2d2* allele and generated a product of the expected size with the *herp* and *cdc 14* primers (suppl. Fig. 4). The corresponding heterozygous flies from stocks 4 and 6 yielded PCR products with both primer combinations. As a control, the heterozygous *r2d2* mutant (suppl. Fig. 4, lane 2) employed in the first crossing step (suppl. Fig. 3, P) and the wild type *w^1118^* stock were verified. Taken together, genomic PCR allowed the identification of stably balanced homozygous and heterozygous *r2d2* mutants.

Supplementary Table 1: Primer Sequences

| r2d2 mutant | herp_s | ACCGACACACCTATGAATCC |
| --- | --- | --- |
|  | r2d2_as | AACAGCGGCAAACCTTCTTA |
|  | cdc14_as | ACGAGAGAGCGCTCTATCAA |
| loqsko mutant | loqs_s | CGCTCATCGACAAGCTGAT |
|  | loqs_as | GAGCAGGCGATCGTAAAGAG |
| blood | blood_s | GCAAAGAAAGCCGAATACCA |
|  | blood_as | CCGGTGGAATCCTTTATCCT |
| copia | copia_s | AGCAAACAACCCCTCATGTC |
|  | copia_as | GCAAACCCAATTTGTCTCGT |
| juan | juan_s | CAATGGGTTGACAACATTCG |
|  | juan_as | CCCAAACAGGTGACCCATAC |
| qbert | qbert_s | CACATATACGGTCGCCTGTG |
|  | qbert_as | GGTCAACGGACAAGGGATTA |
| tinker | tinker_s | CAAGGTCGGCCGAATAATAA |
|  | tinker_as | GACTAGCGAGTCCGATCCAG |
| 1731 | 1731_s | TCGTATGCGGTGATCTGAAG |
|  | 1731_as | CACAACGTGACCCTCTTTCA |
| Gypsy  ([Ghildiyal, Seitz et al. 2008](#_ENREF_43)) | Gypsy_s | CCAGGTCGGGCTGTTATAGG |
|  | Gypsy_as | GAACCGGTGTACTCAAGAGC |
| 297  ([Ghildiyal, Seitz et al. 2008](#_ENREF_43)) | 297_s | AAAGGGCGCTCATACAAATG |
|  | 297_as | TGTGCACATAAAATGGTTCG |
| roo  ([Ghildiyal, Seitz et al. 2008](#_ENREF_43)) | roo_s | CGTCTGCAATGTACTGGCTCT |
|  | roo_as | CGGCACTCCACTAACTTCTCC |
| I-element  ([Ghildiyal, Seitz et al. 2008](#_ENREF_43)) | I-element_s | TGAAATACGGCATACTGCCCCCA |
|  | I-element_as | GCTGATAGGGAGTCGGAGCAGATA |
| mdg1  ([Ghildiyal, Seitz et al. 2008](#_ENREF_43)) | mdg1_s | CACATGTTCTCATTCCCAACC |
|  | mdg1_as | TTCGCTTTTTATATTTGCGCTAC |
| jockey  ([Ghildiyal, Seitz et al. 2008](#_ENREF_43)) | jockey_s | TGCAGTTGTTTCCCCTAACC |
|  | jockey_s | AGTTGGGCAAATGCTAGTGG |
| INE-1  ([Ghildiyal, Seitz et al. 2008](#_ENREF_43)) | INE-1_s | GGCCATGTCCGTCTGTCC |
|  | INE-1_as | AGCTAGTGTGAATGCGAACG |
| blood  ([Ghildiyal, Seitz et al. 2008](#_ENREF_43)) | blood_G_s | TGCCACAGTACCTGATTTCG |
|  | blood_G_as | GATTCGCCTTTTACGTTTGC |
| S-element  ([Ghildiyal, Seitz et al. 2008](#_ENREF_43)) | S-element_s | TGAAAAGCGTCATTCATTCG |
|  | S-element_as | TGTTTCTAGCGCACTCAACG |
| Doc  ([Ghildiyal, Seitz et al. 2008](#_ENREF_43)) | doc_s | GGGTGACTATAACGCCAAGC |
|  | doc_as | GCAAAATCGATCAGGTCTGG |
| 1731  ([Ghildiyal, Seitz et al. 2008](#_ENREF_43)) | 1731_G_s | AGCAAACGTCTGTTGGAAGG |
|  | 1731_G_as | CGACAGCAAAACAACACTGC |
| F-element  ([Ghildiyal, Seitz et al. 2008](#_ENREF_43)) | F-element_s | GCTGGTAGATACCGCTGAGG |
|  | F-element_as | GTAGTCGTCCTCCGTTTTCG |
| 412  ([Ghildiyal, Seitz et al. 2008](#_ENREF_43)) | 412_s | CACCGGTTTGGTCGAAAG |
|  | 412_as | GGACATGCCTGGTATTTTGG |
| NOF  ([Ghildiyal, Seitz et al. 2008](#_ENREF_43)) | NOF_s | AGTTGGACCTGGAATTGTGG |
|  | NOF_as | AATGCACACGGAAGAGGAAC |
| Idefix  ([Ghildiyal, Seitz et al. 2008](#_ENREF_43)) | Idefix_s | AACAAAATCGTGGCAGGAAG |
|  | Idefix_as | TCCATTTTTCGCGTTTACTG |
| Het-A  ([Ghildiyal, Seitz et al. 2008](#_ENREF_43)) | Het_A_s | CGCGCGGAACCCATCTTCAGA |
|  | Het_A_as | CGCCGCAGTCGTTTGGTGAGT |
| piwi | piwi_s | GCATAGGAAGCTGCCATCTC |
|  | piwi_as | TCGTATCTCTCGGGCAGAGT |
| aub | aub_s | AGACCCAGGAATTTGTGCAG |
|  | aub_as | CGAGGCGCGATAACTTTTAG |
| ago3 | ago3_s | CCGCAGAGTTCTCCAAACAT |
|  | ago3_as | GTAGGCATCGATTCGGTCAT |
| gapdh | gapdh_s | AATTTTTCGCCCGAGTTTTC |
|  | gapdh_as | TGGACTCCACGATGTATTCG |
| rp49 | rp49 A2 | ATCGGTTACGGATCGAACA |
|  | rp49 B2 | ACAATCTCCTTGCGCTTCTT |

Supplementary References

1. Park JK, Liu X, Strauss TJ, McKearin DM, Liu Q (2007) The miRNA pathway intrinsically controls self-renewal of Drosophila germline stem cells. Curr Biol 17: 533-538.

2. Forstemann K, Tomari Y, Du T, Vagin VV, Denli AM, et al. (2005) Normal microRNA maturation and germ-line stem cell maintenance requires Loquacious, a double-stranded RNA-binding domain protein. PLoS Biol 3: e236.

3. Liu X, Jiang F, Kalidas S, Smith D, Liu Q (2006) Dicer-2 and R2D2 coordinately bind siRNA to promote assembly of the siRISC complexes. Rna 12: 1514-1520.

Supplementary Legends

Figure S1: Backcrossing schema of *loqs^ko^* mutant in *w^1118^* genetic background.

Virgins of heterozygous *loqs^ko^* mutant rescued with Loqs-PB via P-element insertion were mated with *w^1118^* males (P). The offspring was selected for flies with *loqs^ko^* deletion based on a *mini-white* transgene expressing red eye color pigment serving as a marker gene. The selected offspring was mated with male double balancer flies to obtain balanced autosomes (F1). The third mating of siblings (F2) should produce homozygous and heterozygous *loqs^ko^* mutants (F3).

Figure S2: Fly stock mapping of *loqs^ko^* mutants.

A) Nine fly stocks (lane 1 to 9) resulting from the sibling mating were mapped for *loqs* deletion by PCR. The genomic DNA was isolated according to the Berkeley Drosophila Genome Project protocol. Wild type *loqs* gene and *loqs-PB* were distinguished by size (320 nt and 247 nt, respectively). In lane 10 heterozygous *loqs* mutant applied in the first crossing step was examined as a control to visualize *loqs-PB* and wt *loqs*. Actin served as DNA quality control. B) Flies with *Cy* marker in fly stocks 3 and 7 were tested for heterozygous *loqs^ko^* genotype.

Figure S3: Backcrossing schema of *r2d2^1^* mutant in *w^1118^* genetic background.

A) Virgins of heterozygous *r2d2^1^* mutant balanced over *CyO* were mated with *w^1118^* males (P). The offspring was mated with male double balancer flies to obtain balanced autosomes (F1). As the *r2d2* mutation was not selectable by a marker gene, the third mating of siblings (F2) resulted in four possible combinations of crossing (F3) shown for the relevant chromosome 2. The upper panel demonstrates the resulting genotypes of the offspring while the lower panel presented the corresponding visible phenotypic markers. C) Heterozygous *r2d2* flies balanced over *CyO* were backcrossed with *w^1118^* wt males. The genotype of the offspring for *r2d2* and *loqs^ko^* mutant was depicted for the relevant 2^nd^ chromosome. Flies marked in red were selected as heterozygous controls for further experiments.

Figure S4: Fly stock mapping of *r2d2* mutants.

A) Seven fly stocks (lane 3 to 9) resulting from the sibling mating were mapped for *r2d2* deletion by PCR. Primers were used for *herp* (upstream of *r2d2*), *r2d2* and *cdc14* (downstream of *r2d2*). As control *w^1118^* and heterozygous *r2d2* mutant from first crossing step were analyzed in lane 1 and 2, respectively. Actin served as a positive control. B) Flies with *Cy* marker in fly stocks 4 and 6 were tested for heterozygous *r2d2* genotype.

Figure S5: Verification of β-elimination efficiency.

RNA was isolated from heterozygous and homozygous *loqs-D* and *r2d2^1^* mutants originating from somatic and germline tissue, respectively. They were oxidized with sodium periodate and β-eliminated by raising the pH into high basic range. Each RNA sample, before and after the treatment, was loaded on a 15% Acrylamide-Urea gel and stained with SybrGold. 2S rRNA served as a visible control for β-elimination efficiency due to its high abundance.

**Figure S6: Comparison of read counts in untreated and β-eliminated deep sequencing libraries (somatic RNA samples)**

For this figure, the deep sequencing read counts mapping without mismatch to miRNAs and transposons were determined only for the 21-23 nt size range in order to selectively cover only siRNAs and miRNAs. Each dot represents an individual transposon or microRNA, only the head + thorax RNA samples were analyzed. The top panels depicting the heterozygous controls nicely demonstrate the effect of β-elimination on most miRNAs, which are shifted to the right of the diagonal. Transposon-matching endo-siRNAs, in contrast, are resistant to the treatment.

In the bottom right panel from the homozygous *r2d2* mutant mis-loading of transposon-matching endo-siRNAs into Ago1 can be identified by the less efficient separation from the miRNA population (due to the large spread of the transposon data points). However, as a group the endo-siRNAs are still less sensitive to β-elimination than the miRNAs. If the chemical treatment of the RNA prior to library generation had been limiting, the endo-siRNAs should not be separated from miRNAs at all.

In the loqs-PD mutant on the bottom left, the endo-siRNAs have also become slightly more sensitive to β-elimination. This is consistent with the notion that part of the endo-siRNAs may be loaded into Ago2 via a Loqs-PD containing complex.

Figure S7: Read length distribution of *roo*, *TNFB*, *blood* and *roo* transposon mapping small RNAs in *r2d2* and *loqs-D* mutants.

Reads of each library were mapped to A) *297*, B) *blood* C) *roo* and D) *TNFB* transposon sequence separately and their size distribution was profiled. After normalization to genome matching reads, the counts were expressed as reads per thousand (RPT).

**Figure S8: The length distribution of 412, F-element, doc transposon mapping small RNAs in *r2d2* and *loqs-D* mutants.**

Reads of each library were mapped to A*) F-element*, B) *412* and C) *Doc* transposon sequence separately and their size distribution was profiled. After normalization to total genome matching reads, the size distribution was expressed as reads per thousand (RPT).

Figure S9: Analysis of endo-siRNAs classified in LTRs, LINEs and IRs transposons in *r2d2* and *loqs-D* mutants.

The transposon sequence collection was classified into long terminal repeats (LTRs) and long interspersed elements (LINEs) while DNA transposons were represented by inverted repeats (IRs). Reads of each library were mapped to individual transposons and transposon matching endo-siRNAs were normalized to total genome matching reads (unit: reads per thousand, RPT; note the logarithmic axis scale). The effects of *r2d2* and *loqs-D* mutants were examined during processing and loading within soma and germ line.

**Figure S10: Comparison of endo-siRNA abundance changes with changes in steady-state levels of transposons**

Scatter plots were performed to test the correlation of the change in steady state level of transposon mRNA against the change in loading of endo-siRNAs for specific transposon after deletion of *r2d2* or *loqs-D*. To do so, we compared the fold change of homozygous to heterozygous mutants of deep sequencing data during the loading against qRT-PCR results in soma and germline. Deep sequencing data were normalized to the total genome matching reads while qRT-PCR values were normalized to the average of *rp49* and *gapdh* controls.

**Figure S11: Analysis of ping-pong signature of pilRNAs and piRNAs**

Sequence logo plots of 24-27 nt long sense (+) and antisense (-) transposon matching RNAs were constructed with WebLogo 3.3. The height of symbols within the stack indicated the frequency of each nucleotide A, C, G or U (substituted by T) at this position for the first 15 nt in all analyzed reads. Untreated RNA samples in soma and germ line are depicted in (A) while (B) shows the β-eliminated samples after loading. We examined the overlap between sense- and antisense-matching piRNAs starting from the 5’-end. A peak at 10 nt is characteristic of the mutual cleavage pattern oberserved during ping-pong amplification. To present all genotypes independently of sequencing depth, we scaled each diagram to the sum of all examined overlap sizes (7-14 nt) and displayed the relative proportion of each overlap length.

**Figure S12: Transcript levels of *ago3*, *aub* and *piwi*.**

Somatic RNA isolated from *r2d2* and *loqs-D* mutants was analyzed for expression levels of *ago3*, *aub* and *piwi* by qRT-PCR. The germ line samples known for high abundance of Piwi-family proteins served as positive control. RNA was isolated from soma and germ line of heterozygous and homozygous *r2d2* and *loqs-D* mutants. All Ct values were normalized to the *rp49* control (delta Ct). Note that a low expression results in high Ct-values during qRT-PCR. Normalization to an abundant control does not alter this, therefore a higher ΔCt-value represents lower expression levels. Values are mean ±SD (n=3).


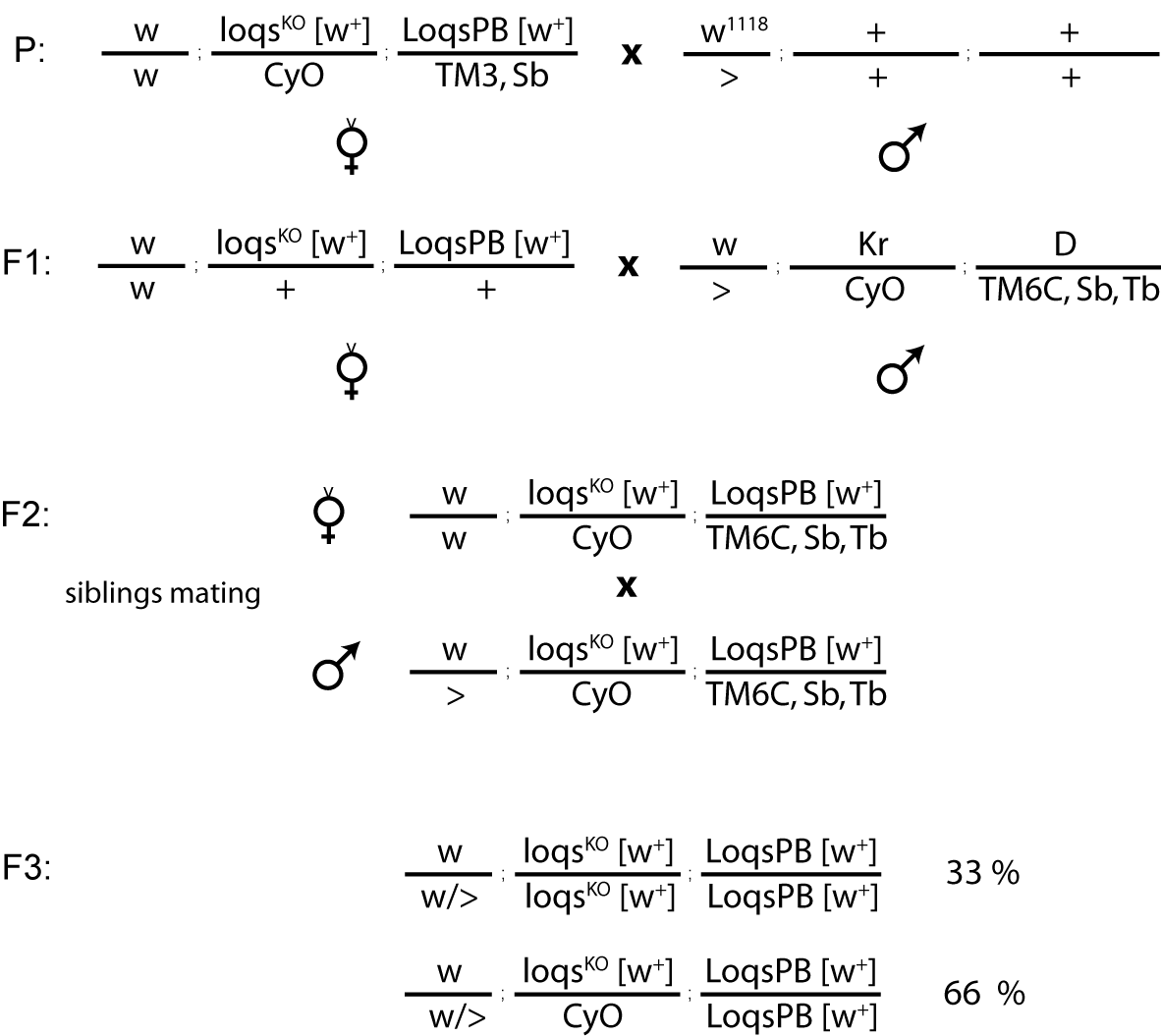


Figure S1: Backcrossing schema of *loqs^ko^* mutant in *w^1118^* genetic background.


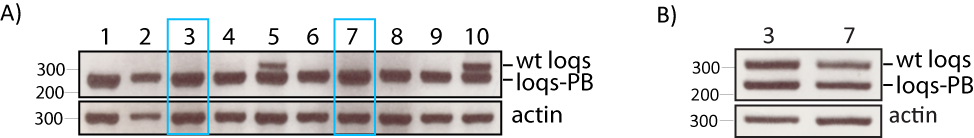


Figure S2: Fly stock mapping of *loqs^ko^* mutants.


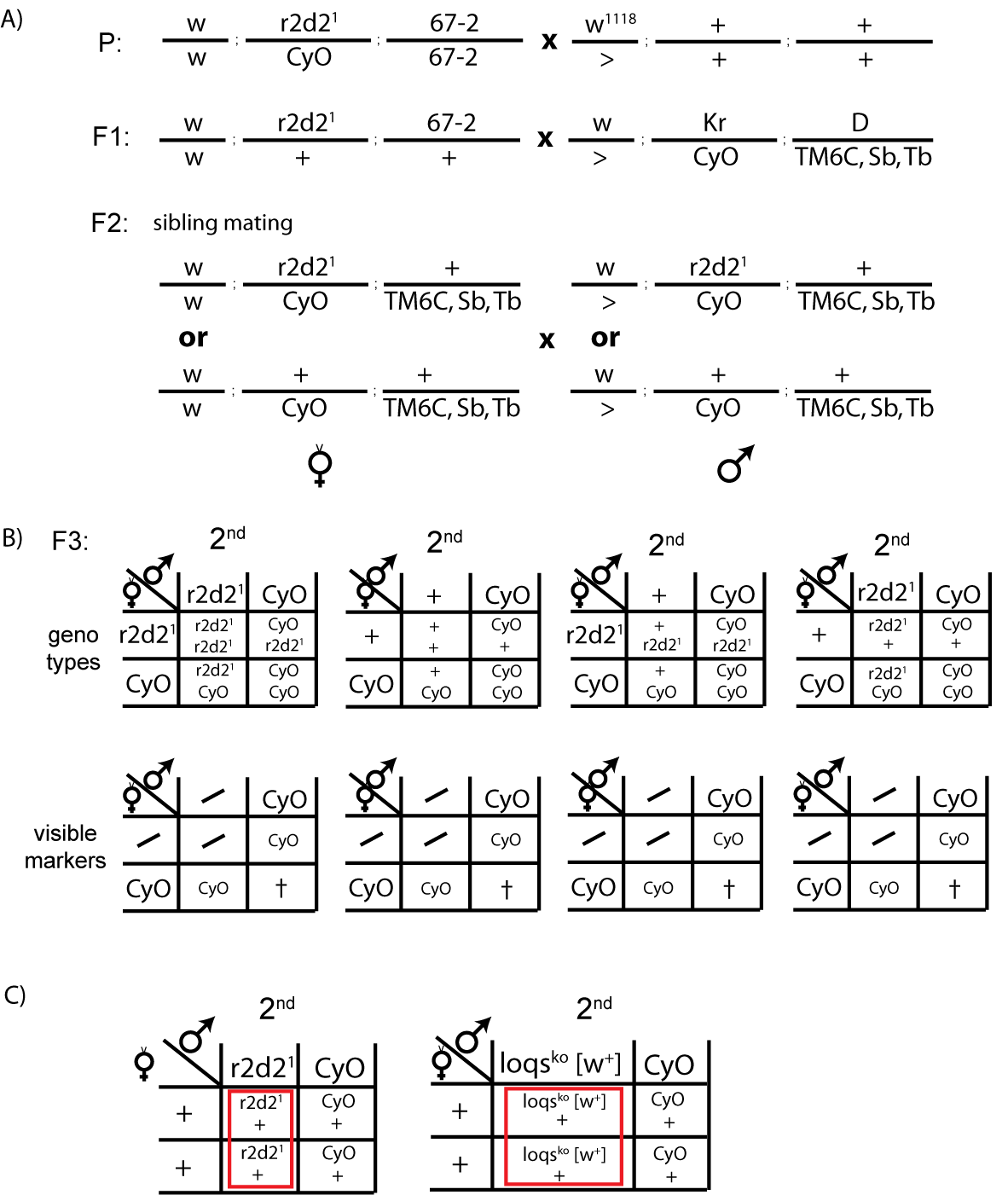


Figure S3: Backcrossing schema of *r2d2^1^* mutant in *w^1118^* genetic background.


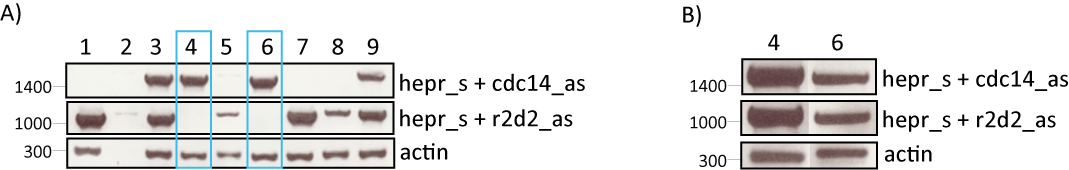


Figure S4: Fly stock mapping of *r2d2* mutants.


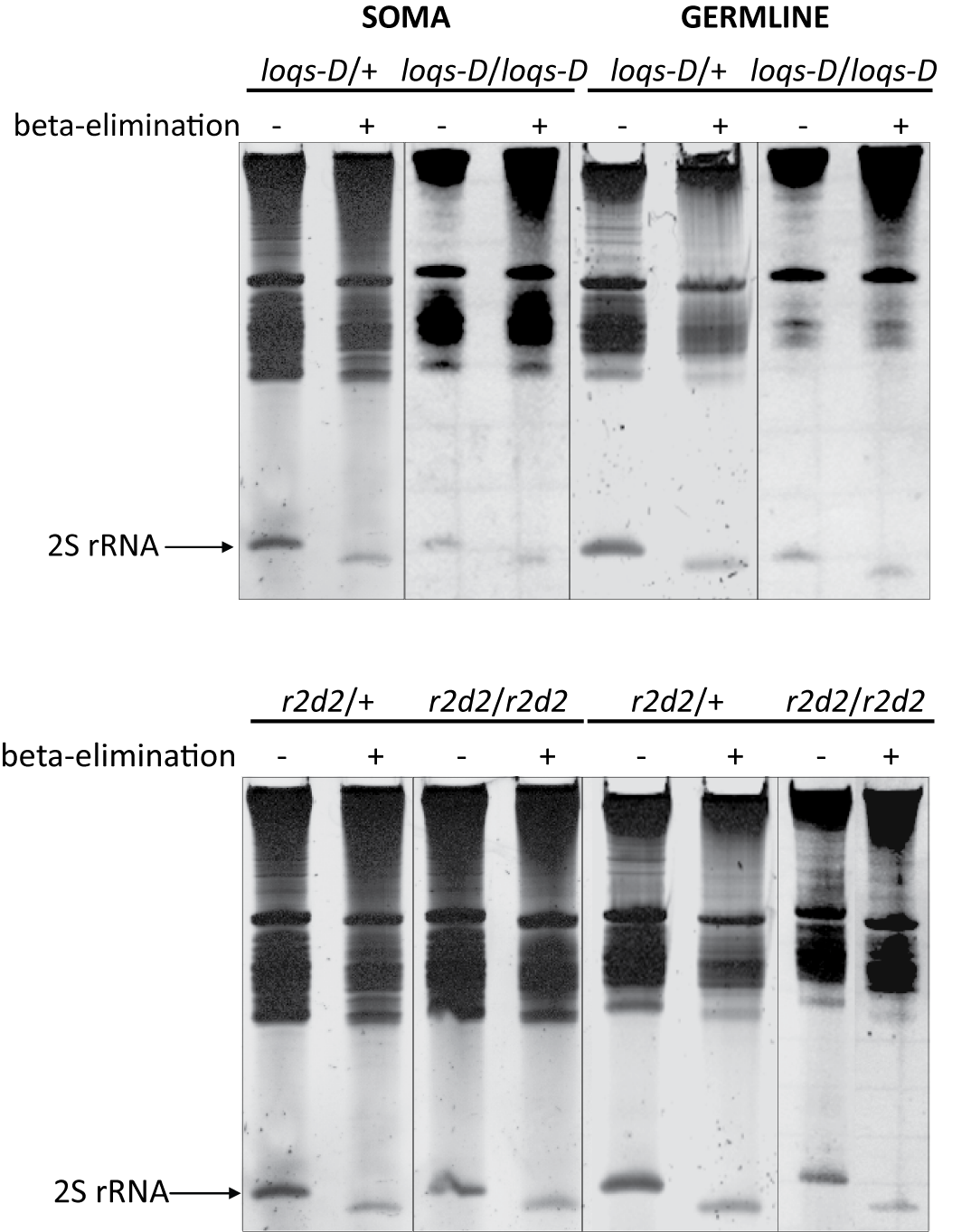


Figure S5: Verification of β-elimination efficiency.


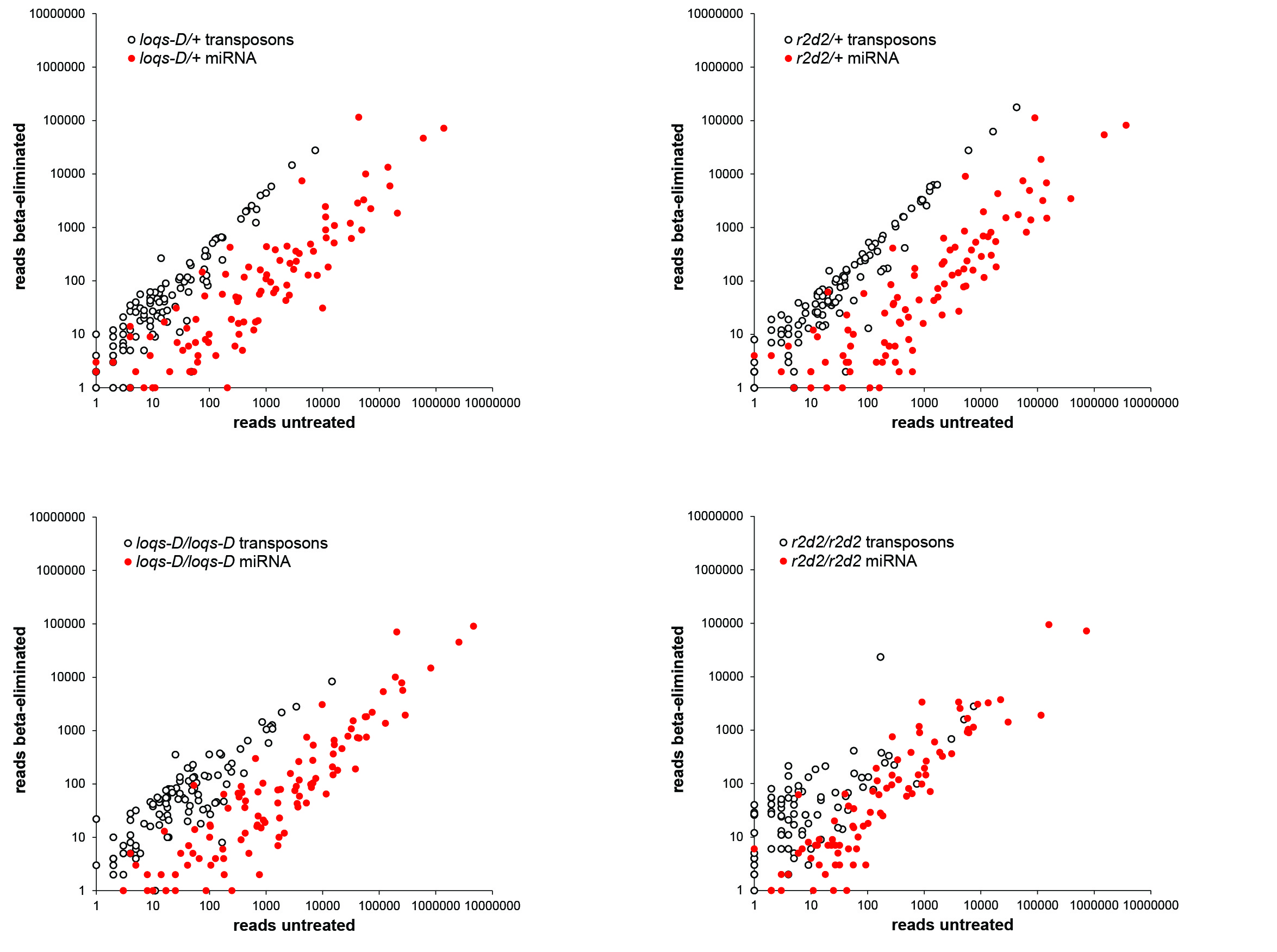


**Figure S6: Comparison of read counts in untreated and β-eliminated deep sequencing libraries (somatic RNA samples)**


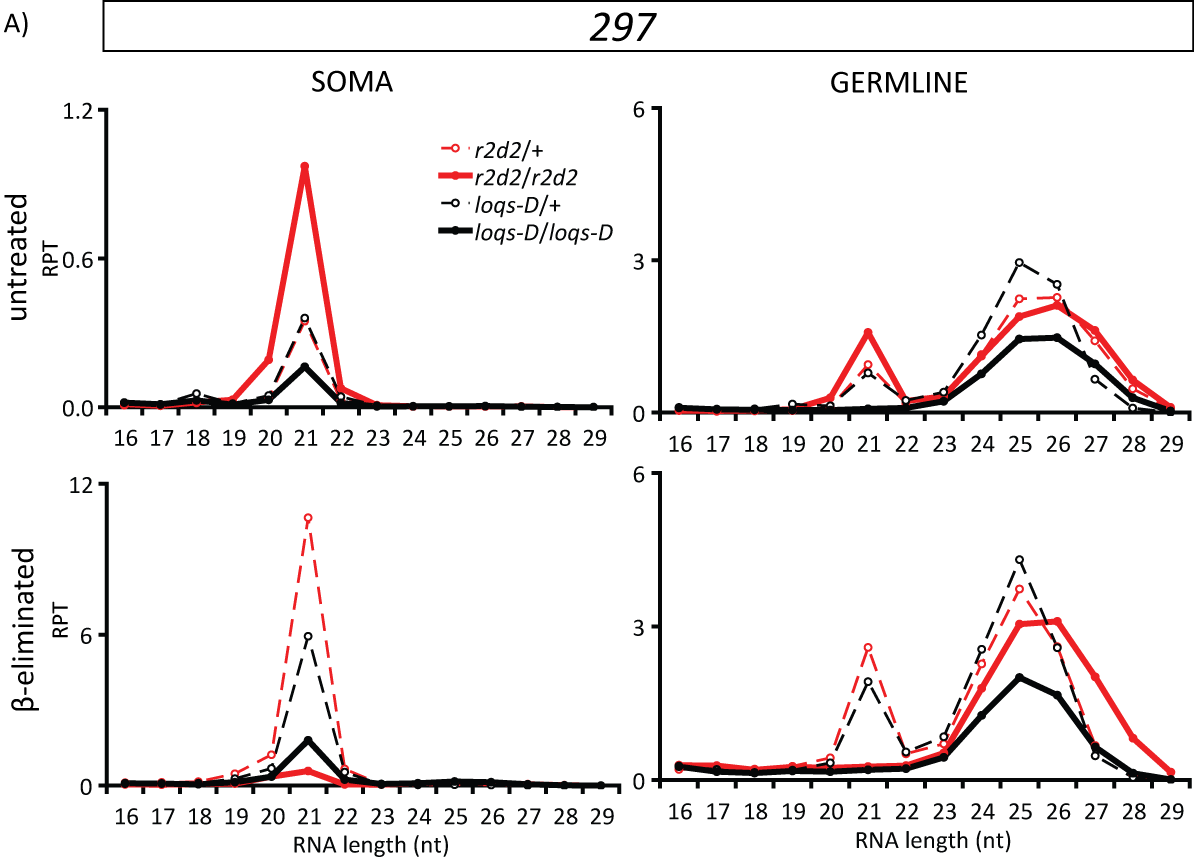


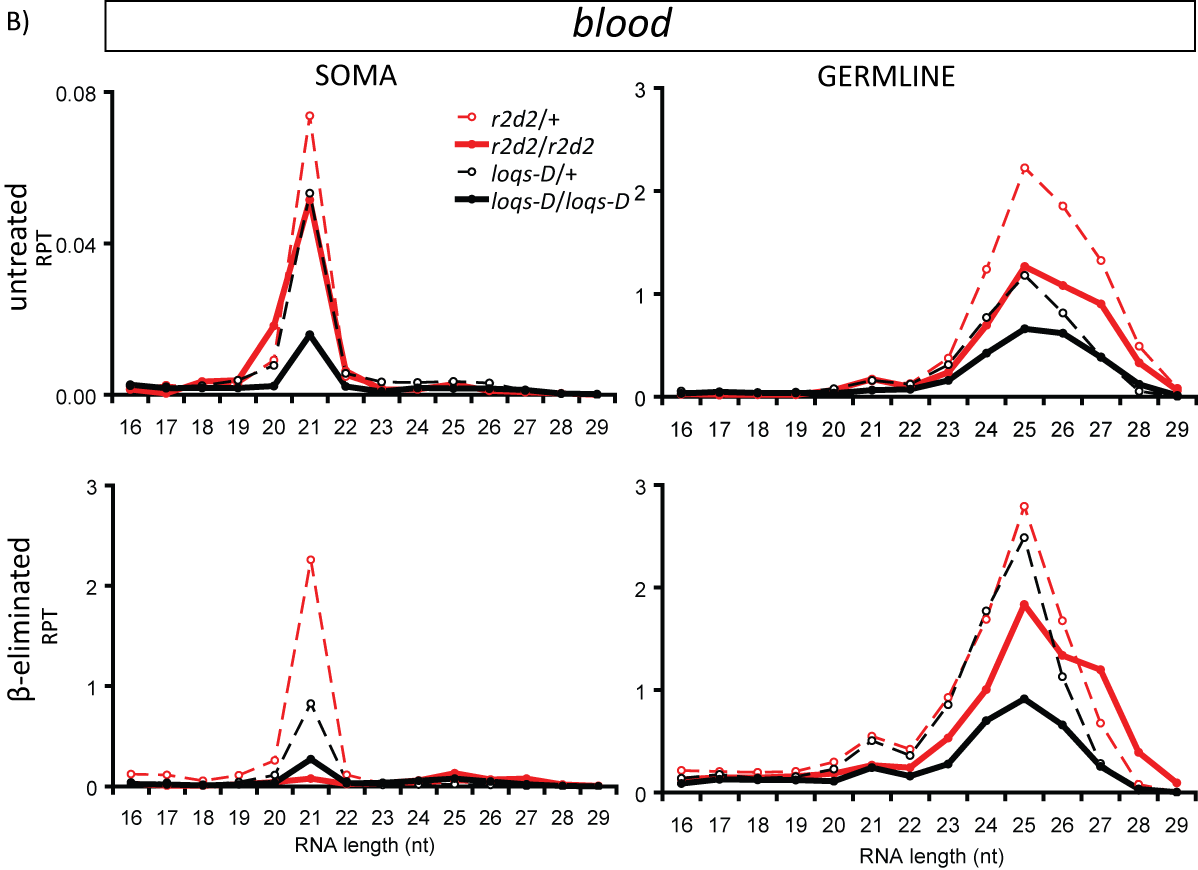


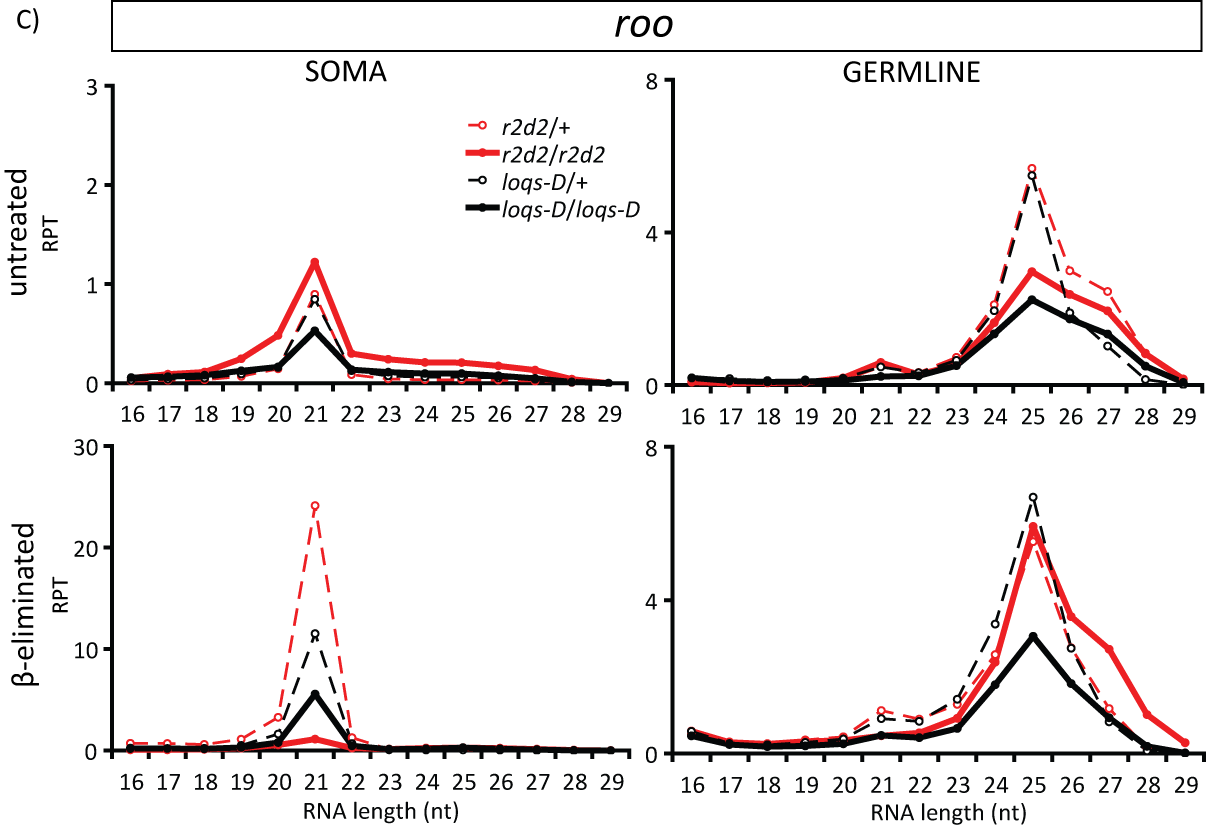

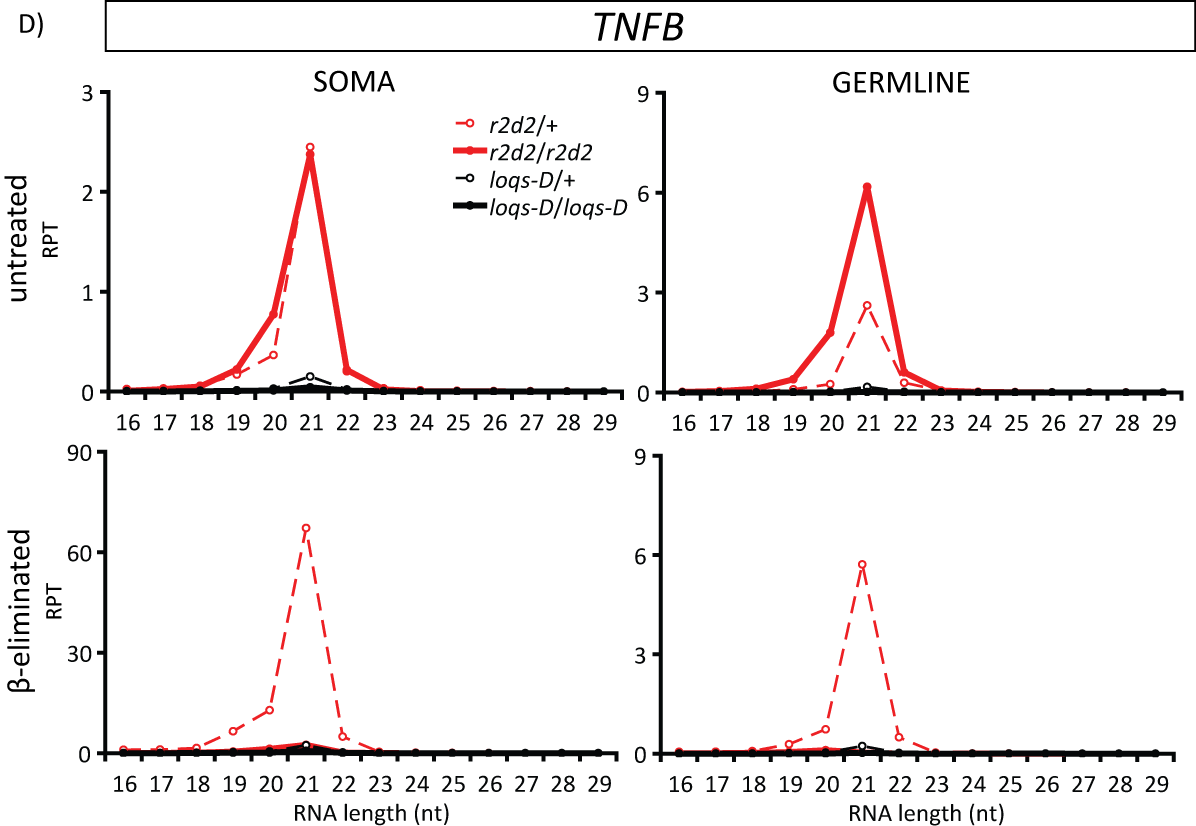


Figure S7: Read length distribution of *roo*, *TNFB*, *blood* and *roo* transposon mapping small RNAs in *r2d2* and *loqs-D* mutants.


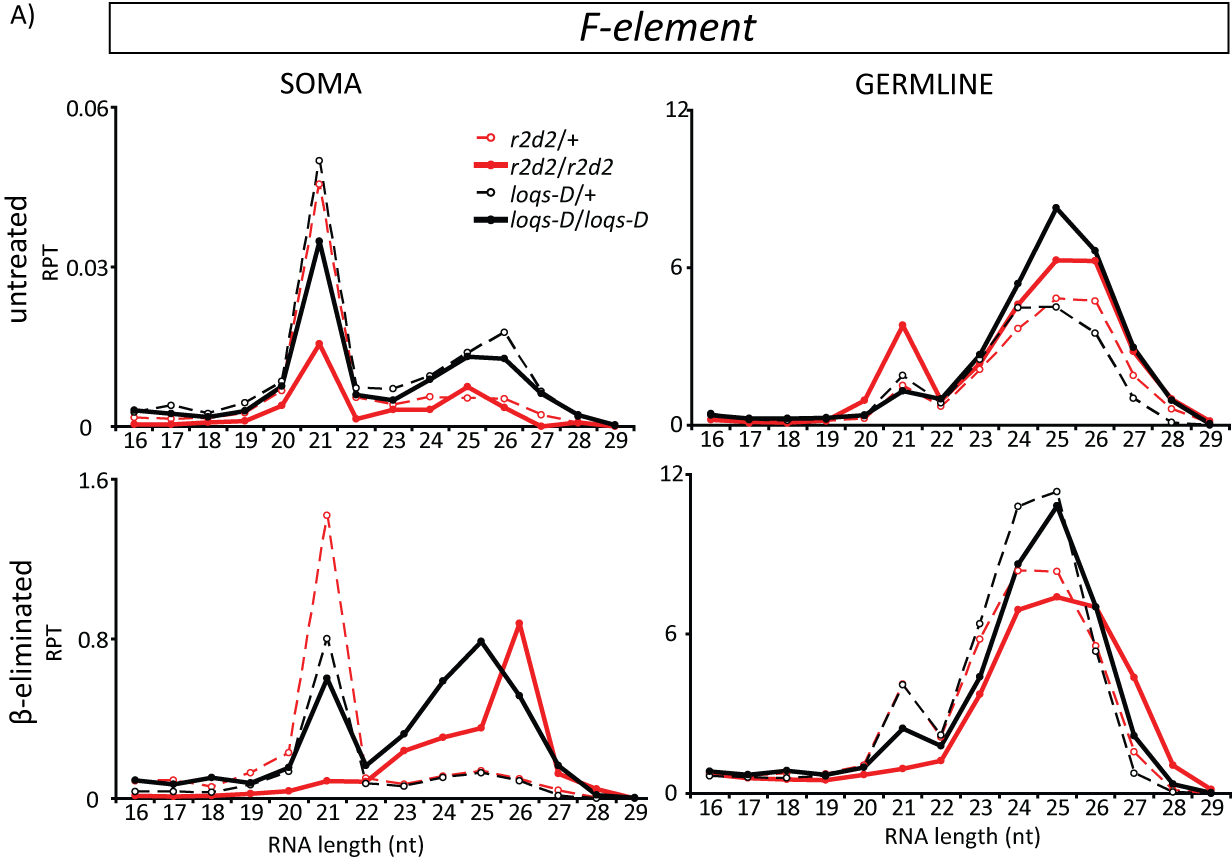


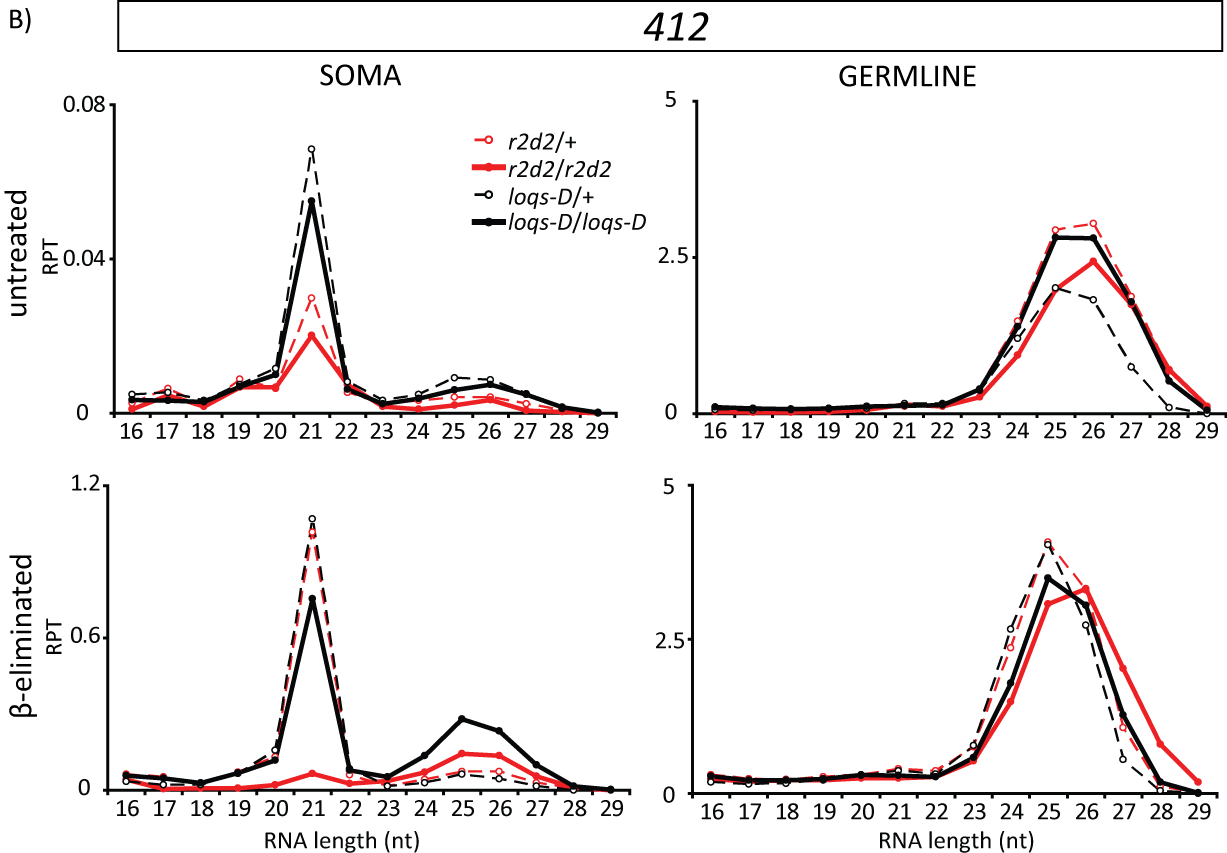


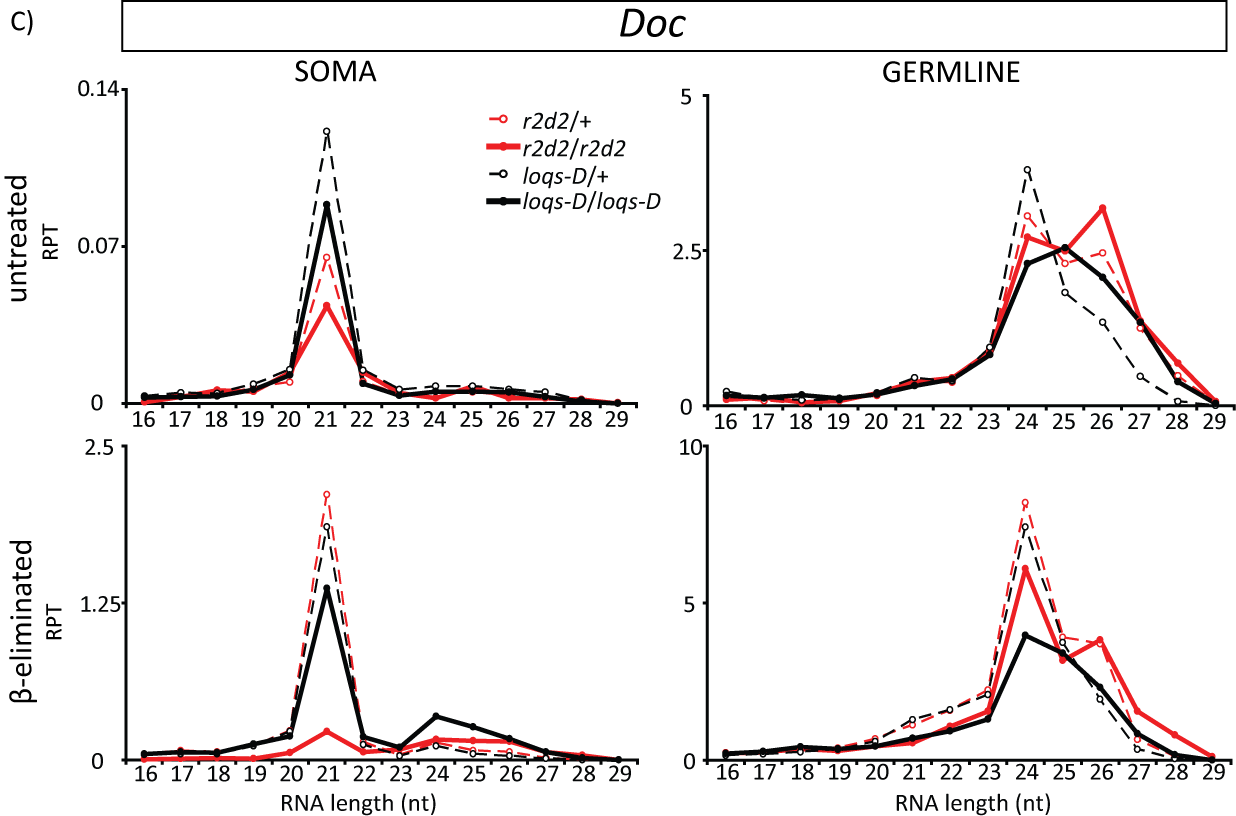


**Figure S8: The length distribution of 412, F-element, doc transposon mapping small RNAs in *r2d2* and *loqs-D* mutants.**


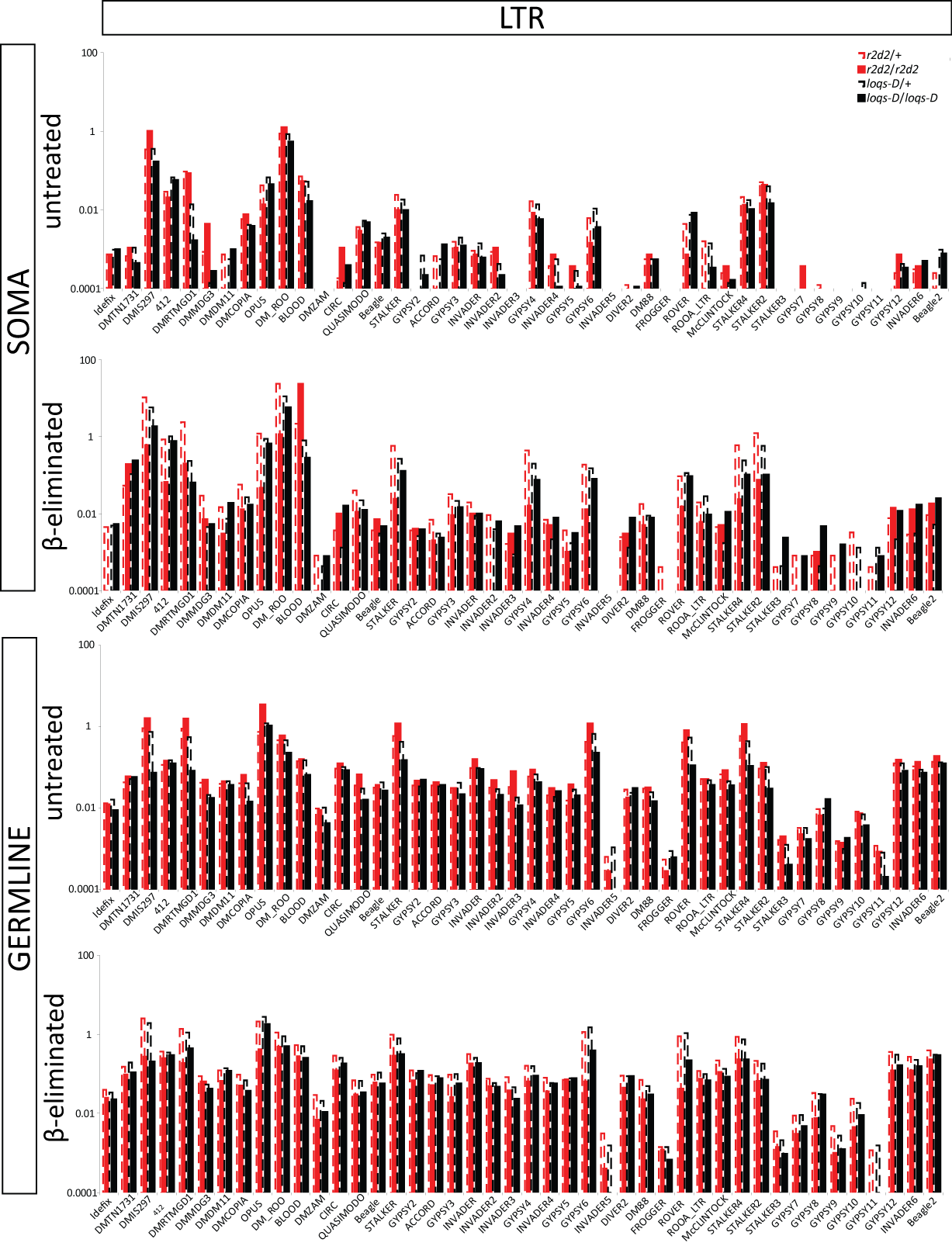


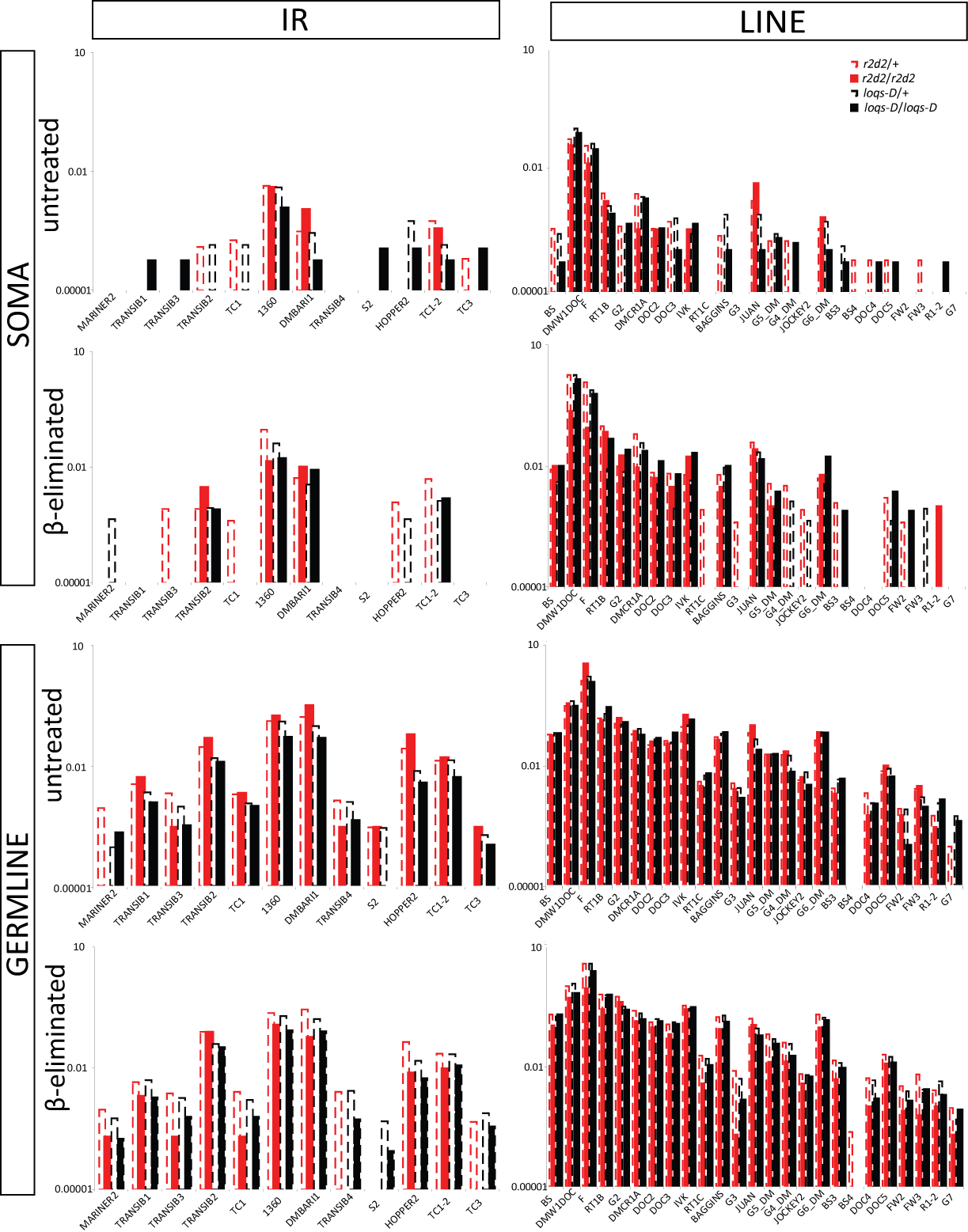


Figure S9: Analysis of endo-siRNAs classified in LTRs, LINEs and IRs transposons in *r2d2* and *loqs-D* mutants.


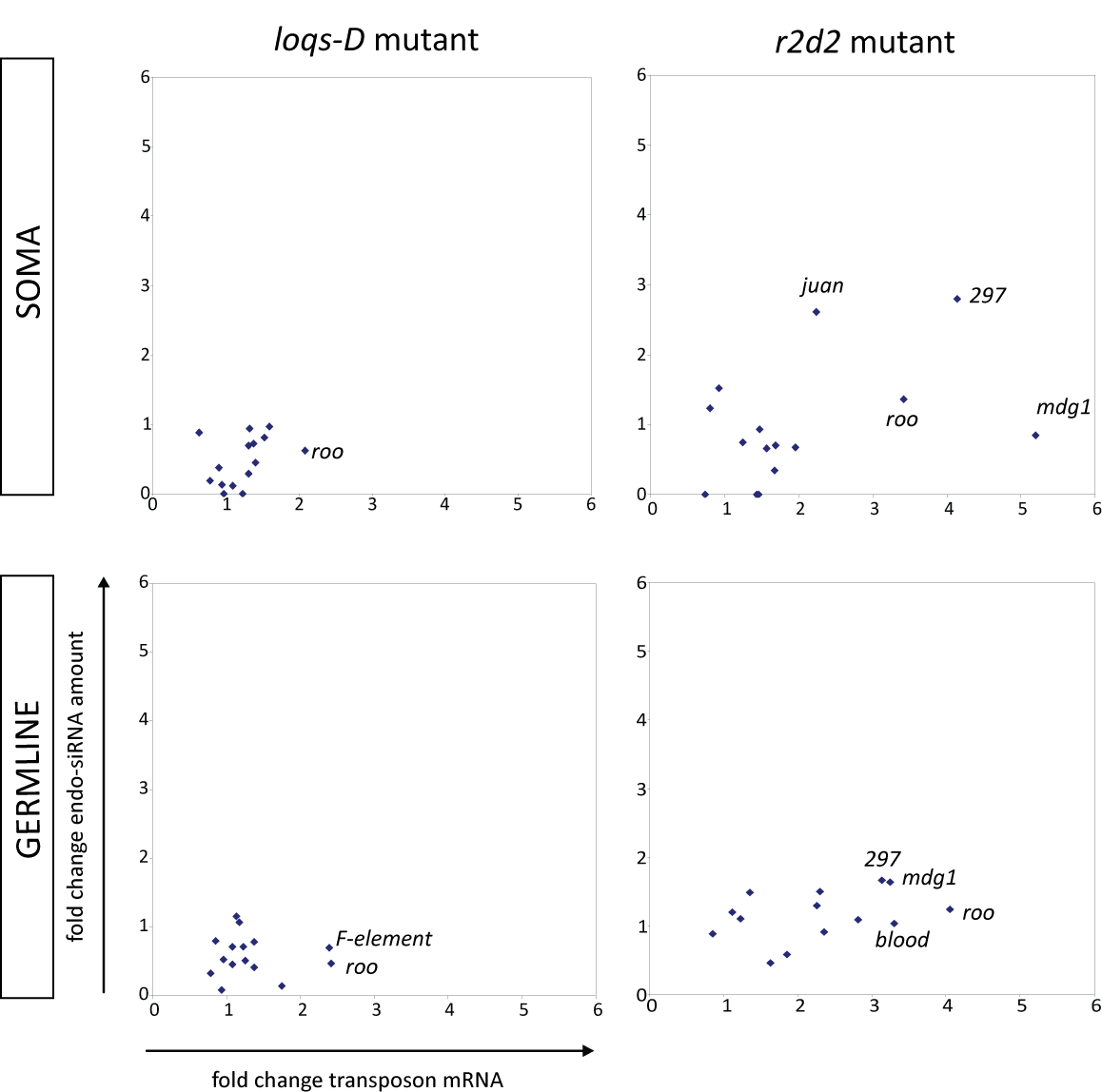

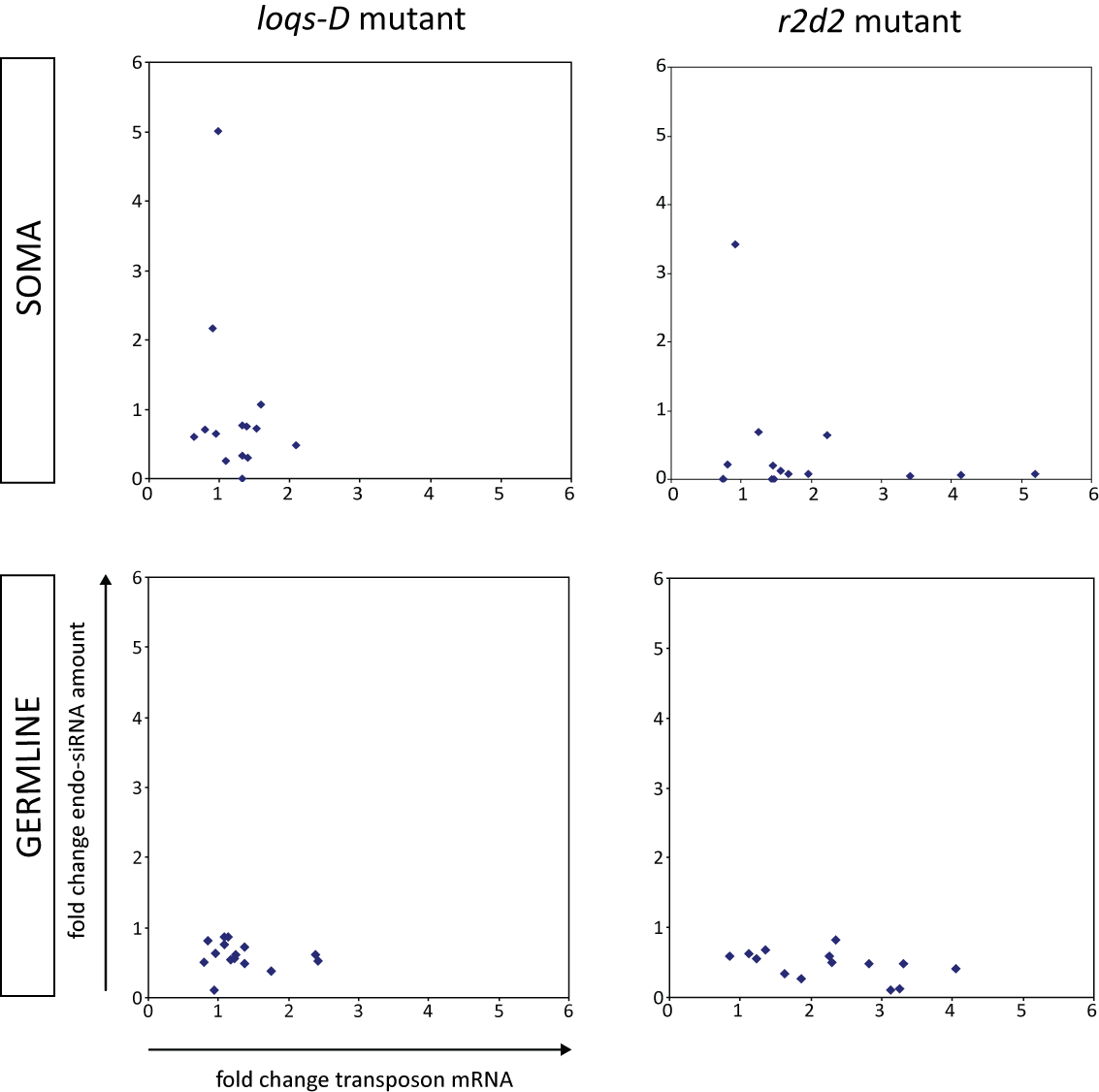


**untreated = processing β-eliminated = loading**

**Figure S10: Comparison of endo-siRNA abundance changes with changes in steady-state levels of transposons**


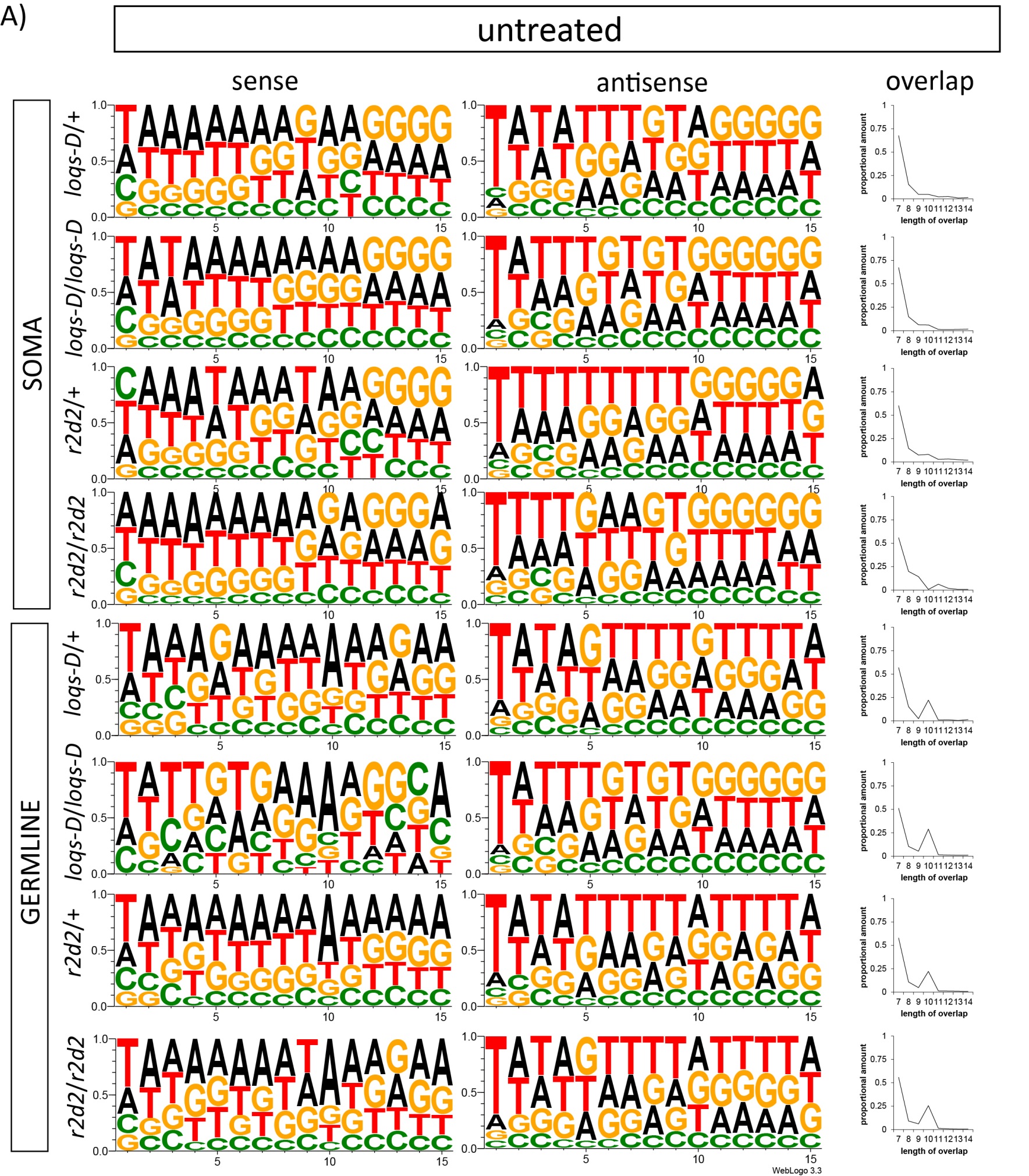


**
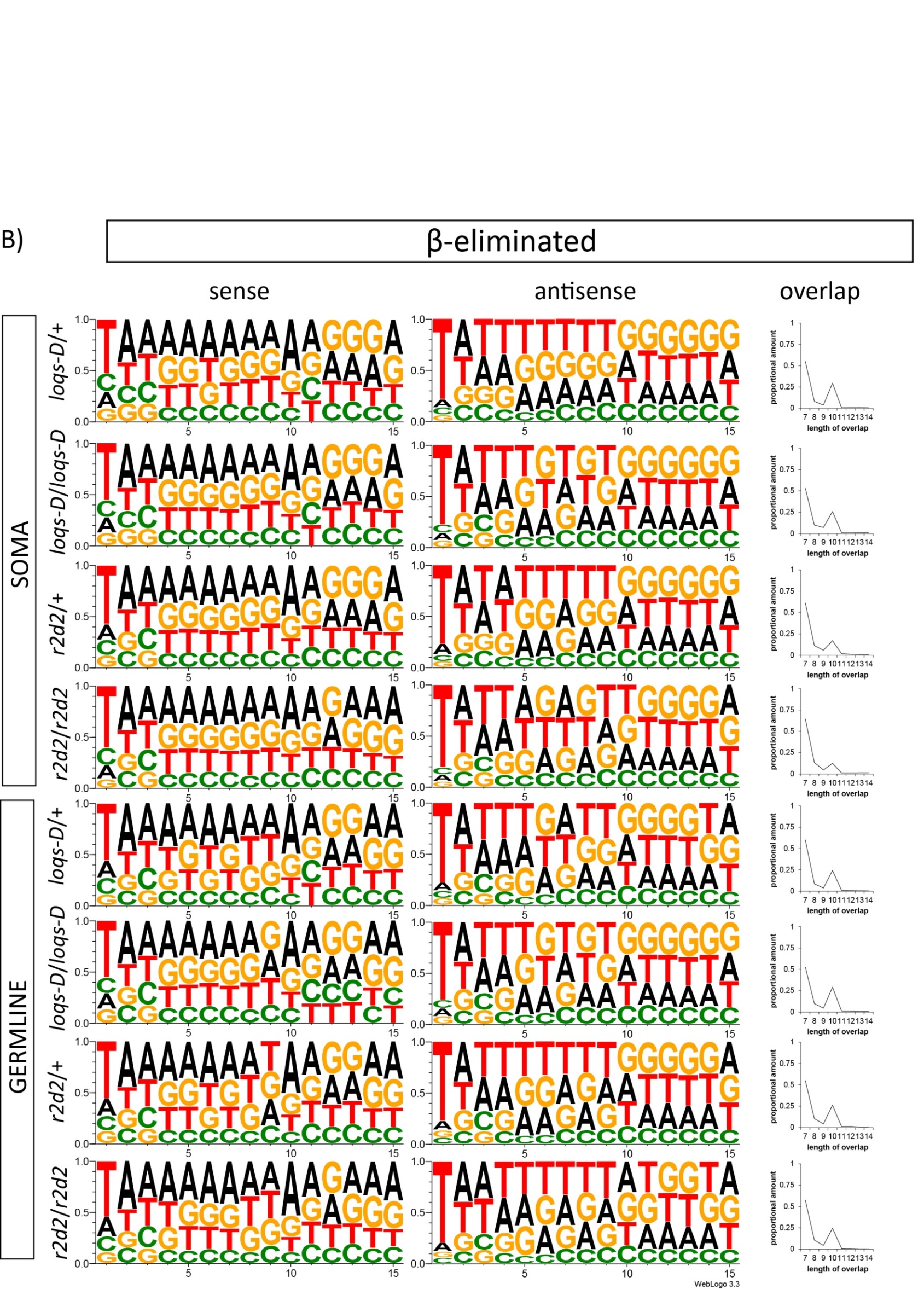
**

**Figure S11: Analysis of ping-pong signature of pilRNAs and piRNAs**


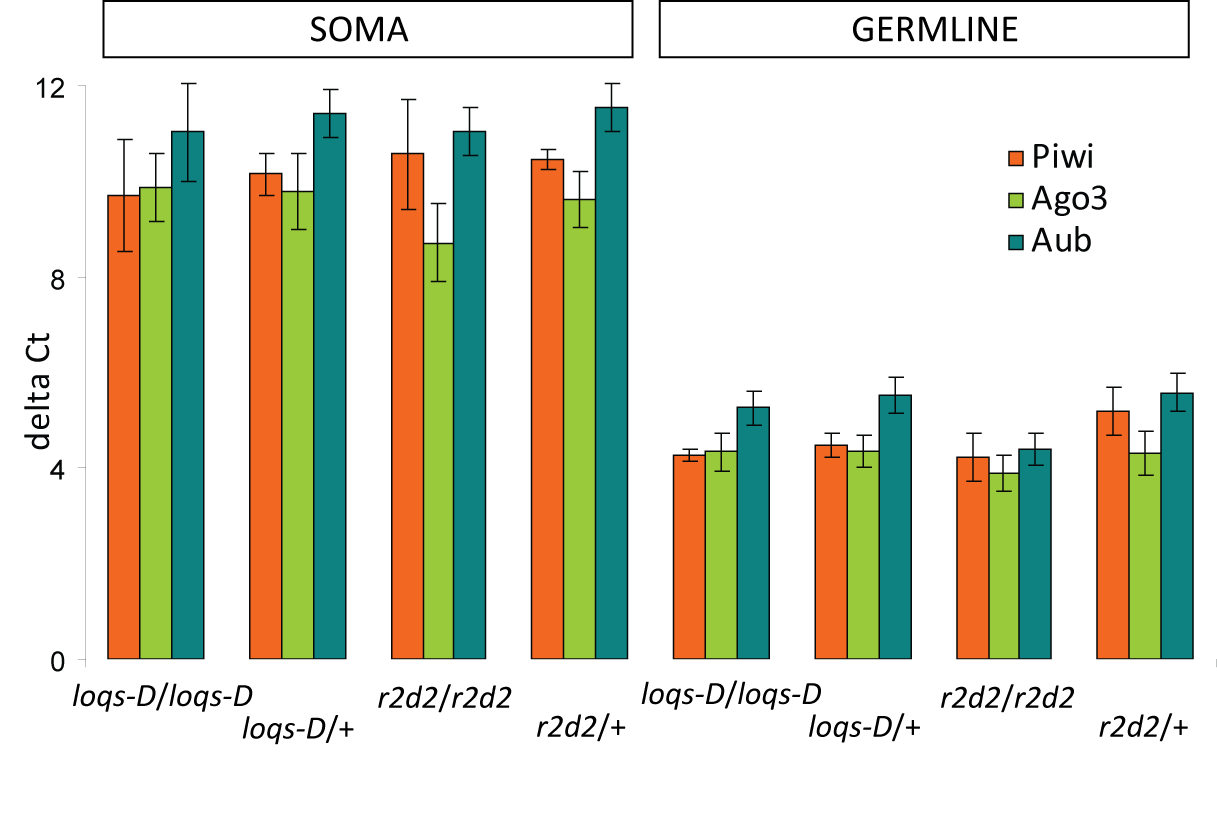


**Figure S12: Transcript levels of *ago3*, *aub* and *piwi*.**
